# MTMR2 promotes invasion and metastasis of gastric cancer via inactivating IFNγ/STAT1 signaling

**DOI:** 10.1186/s13046-019-1186-z

**Published:** 2019-05-21

**Authors:** Lei Jiang, Jun-yan Liu, Yan Shi, Bo Tang, Tao He, Jia-jia Liu, Jun-yan Fan, Bin Wu, Xian-hui Xu, Yong-liang Zhao, Feng Qian, You-hong Cui, Pei-wu Yu

**Affiliations:** 1 Department of General Surgery and Center of Minimal Invasive Gastrointestinal Surgery, Southwest Hospital, Third Military Medical University (Army Medical University), No. 30 Gaotanyan Street, Chongqing, 400038 China; 2 Institute of Pathology and Southwest Cancer Center, and Key Laboratory of Tumor Immunopathology of Ministry of Education of China, Southwest Hospital, Third Military Medical University (Army Medical University), No. 30 Gaotanyan Street, Chongqing, 400038 China

**Keywords:** Myotubularin-related protein 2, Gastric cancer, Invasion, Epithelial- mesenchymal transition (EMT), IFNγ/STAT1 signaling

## Abstract

**Background:**

The aberrant expression of myotubularin-related protein 2 (MTMR2) has been found in some cancers, but little is known about the roles and clinical relevance. The present study aimed to investigate the roles and clinical relevance of MTMR2 as well as the underlying mechanisms in gastric cancer (GC).

**Methods:**

MTMR2 expression was examined in 295 GC samples by using immunohistochemistry (IHC). The correlation between MTMR2 expression and clinicopathological features and outcomes of the patients was analyzed. The roles of MTMR2 in regulating the invasive and metastatic capabilities of GC cells were observed using gain-and loss-of-function assays both in vitro and in vivo. The pathways involved in MTMR2-regulating invasion and metastasis were selected and identified by using mRNA expression profiling. Functions and underlying mechanisms of MTMR2-mediated invasion and metastasis were further investigated in a series of in vitro studies.

**Results:**

MTMR2 was highly expressed in human GC tissues compared to adjacent normal tissues and its expression levels were significantly correlated with depth of invasion, lymph node metastasis, and TNM stage. Patients with MTMR2^high^ had significantly shorter lifespan than those with MTMR2^low^. Cox regression analysis showed that MTMR2 was an independent prognostic indicator for GC patients. Knockdown of MTMR2 significantly reduced migratory and invasive capabilities in vitro and metastases *in vivo* in GC cells, while overexpressing MTMR2 achieved the opposite results. MTMR2 knockdown and overexpression markedly inhibited and promoted the epithelial-mesenchymal transition (EMT), respectively. MTMR2 mediated EMT through the IFNγ/STAT1/IRF1 pathway to promote GC invasion and metastasis. Phosphorylation of STAT1 and IRF1 was increased by MTMR2 knockdown and decreased by MTMR2 overexpression accompanying with ZEB1 down-regulation and up-regulation, respectively. Silencing IRF1 upregulated ZEB1, which induced EMT and consequently enhanced invasion and metastasis in GC cells.

**Conclusions:**

Our findings suggest that MTMR2 is an important promoter in GC invasion and metastasis by inactivating IFNγ/STAT1 signaling and may act as a new prognostic indicator and a potential therapeutic target for GC.

**Electronic supplementary material:**

The online version of this article (10.1186/s13046-019-1186-z) contains supplementary material, which is available to authorized users.

## Background

Gastric cancer (GC) remains the fifth most common cancer type and the third leading cause of cancer-related deaths worldwide [1]. In 2015, approximately 4292,000 new cases of GC were diagnosed and 2814,000 cancer-associated deaths were occurred in China [2]. Despite considerable advances in early detection and treatment, most of the GC patients still die of cancer progression [3]. Invasion and metastasis are the leading causes of GC progression [4]. Therefore, insight into the molecular mechanisms underlying GC invasion and metastasis is urgently needed for developing more effective treatments.

Myotubularin-related protein 2 (MTMR2) is a member of phosphoinositide lipid phosphatase family, which is involved in the regulation of many cell biological processes, such as endocytosis, membrane trafficking, cytokinesis, cell proliferation, and differentiation [5–7]. MTMR2 mutation causes Charcot-Marie-Tooth type 4B1 (CMT4B1), a recessive demyelinating neuropathy characterized by myelin sheaths folded and severe axonal loss [8]. MTMR2 knock-out mice exhibit CMT4B1-like neuropathy and impaired spermatogenesis [9]. Recently, MTMR2 has been reported to be aberrantly expressed in some tumors [10, 11]. Brenner DR et al. applied novel variant prioritization approaches to identify lung cancer variants in 3128 lung cancer cases and 2966 controls, and found that MTMR2 was associated with increased risk of squamous cell carcinoma [10]. In patients with Sézary syndrome, an aggressive, leukemic cutaneous T-cell lymphoma variant, MTMR2 was involved in rearrangements affecting gene expression [11]. However, the roles, mechanisms and clinical relevance of MTMR2 in GC have not been investigated.

In the present study, we found that MTMR2 was overexpressed in human GC tissues and the expression levels were positively correlated with clinicopathological parameters and reversely correlated with outcomes of the patients. MTMR2 upregulated ZEB1 by inactivated IFNγ/STAT1 signaling to induce epithelial-mesenchymal transition (EMT) and consequently enhanced invasion in vitro and metastases in vivo in GC cells. Our findings suggest that MTMR2 may be a novel onco-protein in GC and act as an independent prognostic indicator as well as a therapeutic target for GC patients.

## Materials and methods

### Patients and specimens

A total of 295 formalin-fixed, paraffin-embedded cancerous and paired adjacent normal tissues were obtained from GC patients who received curative surgery at the Southwest Hospital, Third Military Medical University (Chongqing, China) between January 2007 and December 2010. All patients did not receive any radiotherapy, chemotherapy or immunotherapy before surgery. Tumor stage of the specimens were determined according to the 8th Edition tumor-node-metastasis (TNM) staging classification system of the American Joint Committee on Cancer [12]. Detailed clinicopathologic characteristics of the patients were listed in Additional file 1: Table S1. All the patients had complete clinicopathological records and followed-up information for a period of minimal 5 years. Additional six fresh GC cancerous and matched noncancerous tissues were also obtained from GC patients at Southwest Hospital for western blot analyses. This study was in accordance with Helsinki Declaration and approved by the Medical Ethics Committee of the Southwest Hospital, Third Military Medical University. Written informed consent wherever necessary was obtained from study subjects or their guardians.

### Immunohistochemistry (IHC) analysis

IHC staining was performed with Dako REAL Envision Detection System (Dako, Glostrup, Denmark) as previously described [13]. Paraffin sections were stained for indicated antibodies by IHC, isotype serum was used as negative control. The detail information of antibodies used in the present study were listed in Additional file 1: Table S5. The IHC staining score of each sample was calculated as multiplying the staining intensity by the proportion of the positive staining cells [14]. The staining intensity was graded as follows: 0 (negative), 1 (weak), 2 (moderate) and 3 (strong), and the proportion of the positive staining cancer cells was scored as follows: 1 (1–25%), 2 (26–50%), 3 (51–75%) and 4 (76–100%). The IHC score of 6 was determined as the optimal cutoff value analyzed by using the statistical software X-tile [15] (Yale University School of Medicine, New Haven, CT, USA) (Additional file 1: Figure S1A) and SPSS 19.0 software (IBM SPSS Inc., Chicago, USA) (Additional file 1: Figure S1B). Consequently, samples with score ≥ 6 were categorized as MTMR2^high^, otherwise were defined as MTMR2^low^. The IHC score was performed by two pathologists independently in a blinded manner.

### Cell culture

The immortalized gastric epithelium cell line (GES-1) and human GC cell lines SGC7901, BGC823, MKN-45 and MGC803 were purchased from Cell Bank of the Chinese Academy of Sciences (Shanghai, China). AGS was purchased from the American Type Culture Collection. AGS was cultured in F12K medium containing 10% fetal bovine serum (FBS) (Gibco, USA), other cells were cultured in RPMI 1640 (HyClone, USA) containing 10% FBS. All the cells were tested and confirmed negative for mycoplasma contamination using EZ-PCR Mycoplasma Test Kit (Bioind, Israel), and cultured at 37 °C in 5% CO2 and 100% humidity.

### MTMR2 knockdown and overexpression

To knock-down MTMR2 in GC cells, the lentivirus particles containing shRNAs targeted MTMR2 (named sh-MTMR2) and a non-targeting scrambled RNA (named mock) (Additional file 1: Table S2) were obtained from Shanghai Genechem Co., Ltd. (Shanghai, China). For MTMR2 overexpression in GC cells, full-length human MTMR2 cDNA (NM_016156.5) was constructed into a lentiviral expression vector (named over-MTMR2), and empty vector was used as the control (named control) (Shanghai Genechem Co., Ltd). The lentivirus transfection was conducted as previously described [13]. Stably transfected GC cells were selected using puromycin (2 μg/mL). qRT-PCR and western blot analyses were performed to confirm the efficiency of MTMR2 knockdown and over-expression at mRNA and protein levels, respectively.

### siRNA transfection

The sequences of STAT1 siRNA, ZEB1 siRNA, IRF1 siRNA, and Control siRNA were listed in Additional file 1: Table S3 and synthesized by Ruibo Biotechnology Co., Ltd. (Guangzhou, China). GC cells were seeded into 24-well plates and transfected with indicated siRNA or control using Lipofectamine™ 3000 transfection reagent (Invitrogen, CA, USA) according to the manufacturer’s instructions.

### RNA extraction and quantitative real-time PCR (qRT-PCR)

Total RNA was extracted using TRIzol reagent (Invitrogen, CA, USA). Reverse transcription was performed using commercial kit (Takara, Tokyo, Japan). qRT-PCR reactions were performed using SYBR Premix Ex Taq II (Takara, Tokyo, Japan) according to the manufacturer’s protocol. The primer sequences used for qRT-PCR were listed in Additional file 1: Table S4. Relative mRNA expression of the target gene against β-actin was calculated using the 2^-ΔΔCt^ method [16]. All the experiments were performed in triplicates.

### Western blot

Western blot analysis was performed as described previously [13]. The total protein from cell lines and tissues were extracted using RIPA lysis buffer (Beyotime, Shanghai, China) complemented with protease and phosphatase inhibitors (Beyotime, Shanghai, China). The nuclear and cytoplasmic proteins were extracted using a Nuclear and Cytoplasmic Extraction Kit (Thermo Fisher Scientific, Waltham, MA, USA) according to the manufacturer’s instructions. Protein concentrations of cellular or nuclear extracts were determined using BCA Assay Kit (Thermo Fisher Scientifc, Waltham, MA, USA). The detail information of antibodies used in the present study were listed in Additional file 1: Table S5. β-actin was used as loading control. The proteins were visualized using enhanced chemiluminescence Western blot detection system (Bio-Rad, Hercules, CA, USA) according to the manufacturer’s instructions. Then proteins were visualized with SuperSignal West Femto Maximum Sensitivity Substrate (ECL, Thermo) and detected by a ChemiDocXRS system (BioRad). All of the experiments were performed three times independently.

### Migration and invasion assays

Transwell migration and invasion assays were conducted as previously described [13]. For invasion assay, the upper compartment of the transwell chamber (8 μm pore size, 24 wells, Millipore, Billerica, USA) were precoated with 10 μL of diluted Matrigel (Matrigel and RPMI 1640, 1:3, v/v, BD Biosciences, Sparks, USA). Cells were suspended in serum-free medium and seeded into upper chambers at a density of 3 × 10^4^ and 5 × 10^4^ cells for migration and invasion assays, respectively. To examine the effect of signaling agonists and inhibitors on the invasiveness of GC cells, the Matrigel-transwell system were cultured with or without IFNγ (50 ng/mL, R&D Systems, Minnesota, USA), STAT1 siRNA (40 nmol/L), JAK inhibitor I (15 ng/mL, Santa Cruz Biotechnology), ZEB1 siRNA (50 nmol/L), and IRF1 siRNA (50 nmol/L). RPMI-1640 medium containing 10% FBS and indicated inhibitors or agonists were added to lower chamber (600 μL/well). After incubation for 24 h, the non-invasive cells on the top surface of upper chamber were carefully removed with a cotton swab. Cells were fixed, stained and counted. Each experiment was repeated three times.

### Scratch wound healing assay

To evaluate the migration capability of cultured human GC cells, we used the scratch test procedure, as previously described [13]. Briefly, differently treated GC cells were seeded in 6-well culture dishes and grown until to 90% confluence. After pretreatment with 10 μg/mL mitomycin C (Sigma Chemical, MO, USA) for 2 h, the wounds were scratched with a 200 μL pipette tip in the confluent monolayer. Suspension cells were removed by washing with phosphate buffer saline (PBS), remaining cells were incubated in fresh RPMI-1640 medium without FBS. The wound closures were visualized at 0 and 24 h under a microscope (Olympus, Japan). The migration rate of the cells was quantified according to the equation: [(wound length at 24 h) - (wound length at 0 h)] / (wound length at 0 h) × 100.

### Intraperitoneal metastasis assay

Female BALB/c nude mice (5 weeks old) with a mean body weight of 20 g were purchased from the Experimental Animal Center of Third Military Medical University (Chongqing, China), and the intraperitoneal metastasis model was established as previously described [13]. Briefly, differently treated GC cells were suspended in PBS, and intraperitoneally injected into nude mice at 1 × 10^5^ cells/100 μL/mouse. After 4 weeks, the mice were sacrificed and the number of intraperitoneal tumor nodules were counted. The animal experiments were conducted in accordance with institutional guidelines and approved by the Animal Ethics Committee of Southwest Hospital, Third Military Medical University.

### Luciferase reporter assays

Differently treated GC cells were cultured in 24-well plates and transfected with ZEB1 luciferase reporter and Renilla luciferase plasmid using Lipofectamine™ 3000 transfection reagent (Invitrogen, CA, USA). After transfected for 48 h, the luciferase activities of GC cells were conducted using the luciferase reporter assay system (Promega, USA) according to the manufacturer’s instructions. The transcription activities of ZEB1 promoter were normalized against the co-transfected Renilla luciferase reporter. Three independent experiments were performed.

### Chromatin immunoprecipitation (ChIP) assay

To perform the ChIP assay, four pairs of primers for the ZEB1 promoter were designed and synthesized by Tsingke Biotech Company (Beijing, China). The sequences of the primers were listed in Supplementary Additional file 1: Table S7. ChIP assay was conducted with the Simple ChIP Enzymatic Chromatin IP Kit (magnetic beads; CST, Danvers, MA, USA) according to the manufacture’s instruction. In brief, MGC 803 cells (1 × 10^7^) were fixed with 1% (v/v) formaldehyde to crosslink the proteins and DNA for 10 min at room temperature, followed by treatment with 125 mM glycine for 10 min to quench the crosslinking. The chromatin was sonicated into 200–1000 bp DNA/protein fragments. The lysate was incubated with either IRF1 antibody (#8478, CST) or normal rabbit IgG overnight at 4 °C with rotation. After incubation with ChIP-Grade Protein G Magnetic Beads for 2 h at 4 °C with rotation, the bead-bound immune complexes were then eluted by incubation in 5 mol/L NaCl and Proteinase K at 65 °C for 2 h. Finally, the DNA fragments were purified and amplified by qPCR. The experiments were independently repeated at least three times.

### Statistical analysis

The data were analyzed using SPSS 19.0 software and GraphPad Prism 6.0 software. All data are expressed as the mean ± standard deviation (SD) at least three independent experiments. The comparison of mean between two groups was conducted using Student’s t test. The Pearson χ2 test was used to analyze the association of MTMR2 expression and clinicopathologic parameters. Comparison among multiple groups were analyzed by one-way analysis of variance. Hazard ratios (HRs) and 95% confidence intervals (CIs) were used to estimate the correlation between MTMR2 expression and overall survival (OS) or disease free survival (DFS). OS and DFS curves were plotted using Kaplan-Meier method and compared using log-rank test. Univariable and multivariable Cox proportional hazard regression models were used to identify independent prognostic factors. OS was defined as the time from the time of surgery to the date of death due to any cause or censoring. DFS was defined as the time from surgery to the date of disease recurrence, death or censoring. IPA® software (QIAGEN, Redwood City, https://www.qiagenbioinformatics.com/products/ingenuity-pathway-analysis/) was used to assign differentially expressed genes to known canonical pathways and functional networks. All tests were two-tailed. *P* < 0.05 was considered statistically significant.

## Results

### Elevated MTMR2 is associated with clinicopathologic characteristics of GC and prognosis of the patients

**Fig. 1:**
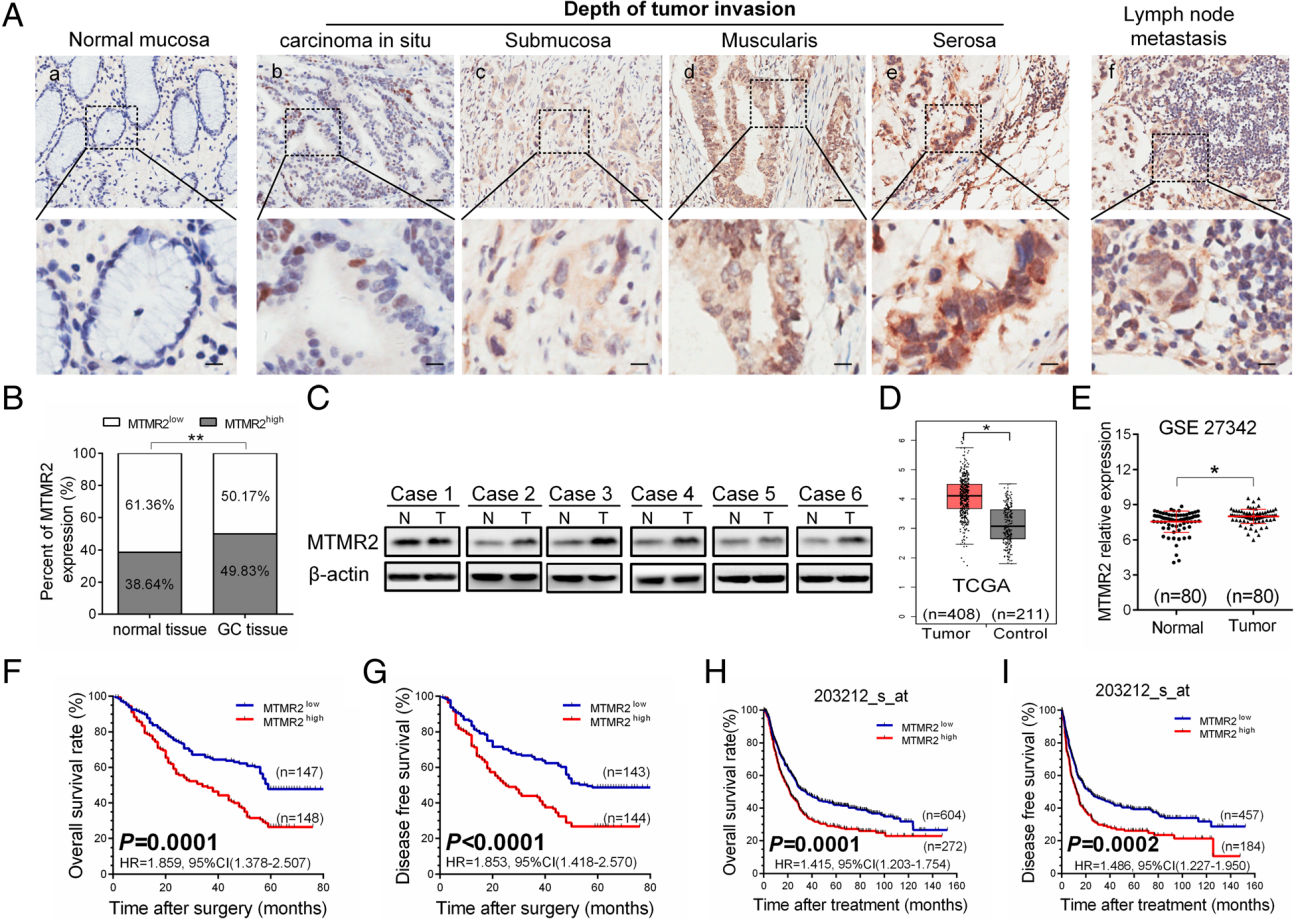
MTMR2 is highly expressed in GC tissues and correlated with the clinicopathological features and the outcome of the patients. **a** Representative images of MTMR2 IHC staining in normal mucosa tissues, GC tissues and metastatic lymph node (scale bar = 50 μm). (Aa) Absence of MTMR2 expression in normal gastric mucosa. (Ab-e) The intensity of MTMR2 staining was increased with the tumor invasion depth (b: carcinoma in situ; c: submucosa; d: muscularis; d: serosa). (Af) Strong staining of MTMR2 was observed in metastatic lymph node. **b** The percentage of MTMR2^high^ in GC tissues was significantly higher than that in adjacent normal tissues; ** *P* < 0.01. **c** In six pairs of fresh surgical GC specimen, the expression level of MTMR2 protein detected by western blot in tumor tissues (T) was higher than that in adjacent normal tissues (N). **d** Data in the TCGA database showed higher MTMR2 expression in GC tumor tissues compared to adjacent normal tissues; * *P* < 0.05. **e** Analysis of GEO GSE 27342 showed a significantly higher MTMR2 expression in GC tissues compared to adjacent normal tissues. **f**, **g** Kaplan-Meier analysis of 295 GC patients indicated that high MTMR2 expression was associated with shorter overall survival (OS) (**f**) and disease free survival (DFS) time (**g**). **h**, **i** Kaplan-Meier analysis of GC patients from dataset 203212_s_at in Kaplan-Meier plotter indicated shorter OS (**h**) and DFS time (**i**) for patients with MTMR2^high^

The expression of MTMR2 in 295 GC cancerous and paired adjacent normal tissues were examined by IHC staining. MTMR2 staining was located in the cytoplasm and/or nucleus of GC cells (Fig. 1a). MTMR2 staining was low or absent in adjacent normal gastric mucosa (Fig. 1Aa), but was high in tumor tissues and metastatic lymph node (Fig. 1Ab-f). Moreover, the intensity of MTMR2 staining in tumor tissues was increased with invasion depth (Fig. 1Ab-e). MTMR2^high^ was more frequent in cancerous tissues (147/295; 49.83%) than that in adjacent normal tissues (114/295; 38.64%) (*P* < 0.001) (Fig. 1b). In six pairs of fresh GC specimens, the expression levels of MTMR2 at protein level in cancerous tissues (T) were higher than that in normal gastric tissues (N) (Fig. 1c). The analysis of MTMR2 RNA-seq data containing 408 GC and 211 normal tissues in TCGA database showed that the mRNA level of MTMR2 expression in GC cancerous tissues was significantly higher than that in normal tissues (*P* < 0.05, Fig. 1d). A similar result was obtained from the analysis of GSE27342 datasets (*P* = 0.0326, Fig. 1e). These results indicate that MTMR2 is significantly upregulated in GC tissues.

We next evaluated the relationship between MTMR2 expression and clinicopathological features in GC. The expression levels of MTMR2 were significantly correlated with T stage (*P* = 0.004), N stage (*P* = 0.000), and TNM stage (*P* = 0.032) (Table 1, Additional file 1: Figure S2A-C), but not with the sex (*P* = 0.396), age (*P* = 0.313), tumor location (*P* = 0.524), histological grade (*P* = 0.172) or tumor size (*P* = 0.056) (Table 1). The mRNA expression level of MTMR2 in TCGA database was also associated with TNM stage (Additional file 1: Figure S2D) analyzed by an online tool ualcan (http://ualcan.path.uab.edu) [17]. The prognostic significance of MTMR2 expression in GC was further estimated. In 295 GC patients, 180 cases (61.02%) died within the median (45 months) of follow-up period (range, 1–81 months). Kaplan-Meier survival analysis showed that patients with MTMR2^high^ had shorter OS time (HR = 1.859, 95%CI (1.378–2.507), *P* = 0.0001) (Fig. 1f) and DFS (HR = 1.853, 95% CI =1.418–2.570, *P* < 0.0001) than those with MTMR2^low^ (Fig. 1g). In addition, we found a cohort 203212_s_at in GEO database, which contained 876 GC patients with follow-up information and also showed that the patients with MTMR2 high expression exhibited shorter OS (HR = 1.415, 95%CI =1.203–1.754, *P* = 0.0001) (Fig. 1h) and DFS (HR = 1.486, 95%CI = 1.227–1.950, *P* = 0.0002) (Fig. 1i). The prognostic value of MTMR2 expression was further investigated after stratification by TNM stage. GC patients with MTMR2^high^ had significantly shorter OS (Additional file 1: Figure S3A and B) and DFS (Additional file 1: Figure S3C and D) in our cohort in both early (TNM I and II) and late (TNM III and IV) stages. The cox proportional hazards regression was performed to further reveal the role of MTMR2 in predicting GC prognosis. Multivariate analysis with stepwise selection method identified that MTMR2^high^ was an independent prognostic factors for OS (*P* = 0.004, Table 2, Additional file 1: Figure S4A) and DFS (*P* = 0.006, Table 3, Additional file 1: Figure S4B). These results suggest that MTMR2 may act as a promising prognostic marker for GC patients.

**Table 1:** The relationship between MTMR2 expression and clinicopathological features in patients with GC

Prognostic variables	Number	MTMR2 expression		χ^2^	P
		Low	High		
Sex				0.722	0.396
Male	206	100	106		
Female	89	48	41		
Age(years)				1.016	0.313
< 60	185	97	88		
≥ 60	110	51	59		
Tumor location				0.407	0.524
Proximal+middle	131	63	68		
Distal	164	85	79		
Histological grade				1.868	0.172
G1 + G2	87	49	38		
G3	208	99	109		
Tumor size				3.645	0.056
< 5 cm	200	108	92		
≥ 5 cm	95	40	55		
T stage				8.098	**0.004**
T1-T2	80	51	29		
T3-T4	215	97	118		
N stage				15.493	**0.000**
N0	96	64	32		
N1-N3	199	84	115		
M stage				4.622	**0.032**
M0	276	143	133		
M1	19	5	14		
TNM stage				4.653	**0.031**
I + II	143	81	62		
III + IV	152	67	85

**Table 2:** Univariate and multivariate analyses of OS in patients with GC

Prognostic variables	Univariate OS analysis			Multivariate OS analysis		
	HR	95% CI	*P* value	HR	95% CI	*P* value
Sex	0.819	0.592–1.133	0.228	–	–	–
Age	1.068	0.788–1.446	0.671	–	–	–
Histological grade	0.920	0.670–1.264	0.607	–	–	–
Tumor location	0.752	0.561–1.008	0.057	–	–	–
T stage	2.515	1.728–3.660	0.000	1.675	1.061–2.646	0.027
N stage	2.103	1.502–2.944	0.000	1.310	0.834–2.058	0.241
M stage	4.505	2.734–7.422	0.000	3.052	1.803–5.166	0.000
TNM stage	2.141	1.587–2.890	0.000	1.063	0.668–1.689	0.798
Tumor size	2.187	1.618–2.956	0.000	1.624	1.179–2.237	0.003
MTMR2 expression	1.826	1.356–2.460	0.000	1.829	1.216–2.751	0.004

**Table 3:** Univariate and multivariate analyses of DFS in patients with GC

Prognostic variables	Univariate DFS analysis			Multivariate DFS analysis		
	HR	95% CI	*P* value	HR	95% CI	*P* value
Sex	0.786	0.566–1.093	0.153	–	–	–
Age	1.096	0.808–1.487	0.554	–	–	–
Histological grade	0.928	0.674–1.277	0.645	–	–	–
Tumor location	0.758	0.564–1.019	0.066	–	–	–
T stage	2.457	1.687–3.579	0.000	1.632	1.031–2.584	0.036
N stage	2.066	1.474–2.896	0.000	1.281	0.812–2.019	0.287
M stage	4.491	2.724–7.404	0.000	2.996	1.766–5.084	0.000
TNM stage	2.119	1.566–2.866	0.000	1.072	0.670–1.715	0.771
Tumor size	2.201	1.625–2.981	0.000	1.622	1.174–2.242	0.003
MTMR2 expression	1.866	1.381–2.522	0.000	1.555	1.134–2.131	0.006

### MTMR2 enhances the invasion and metastasis of GC cells

**Fig. 2:**
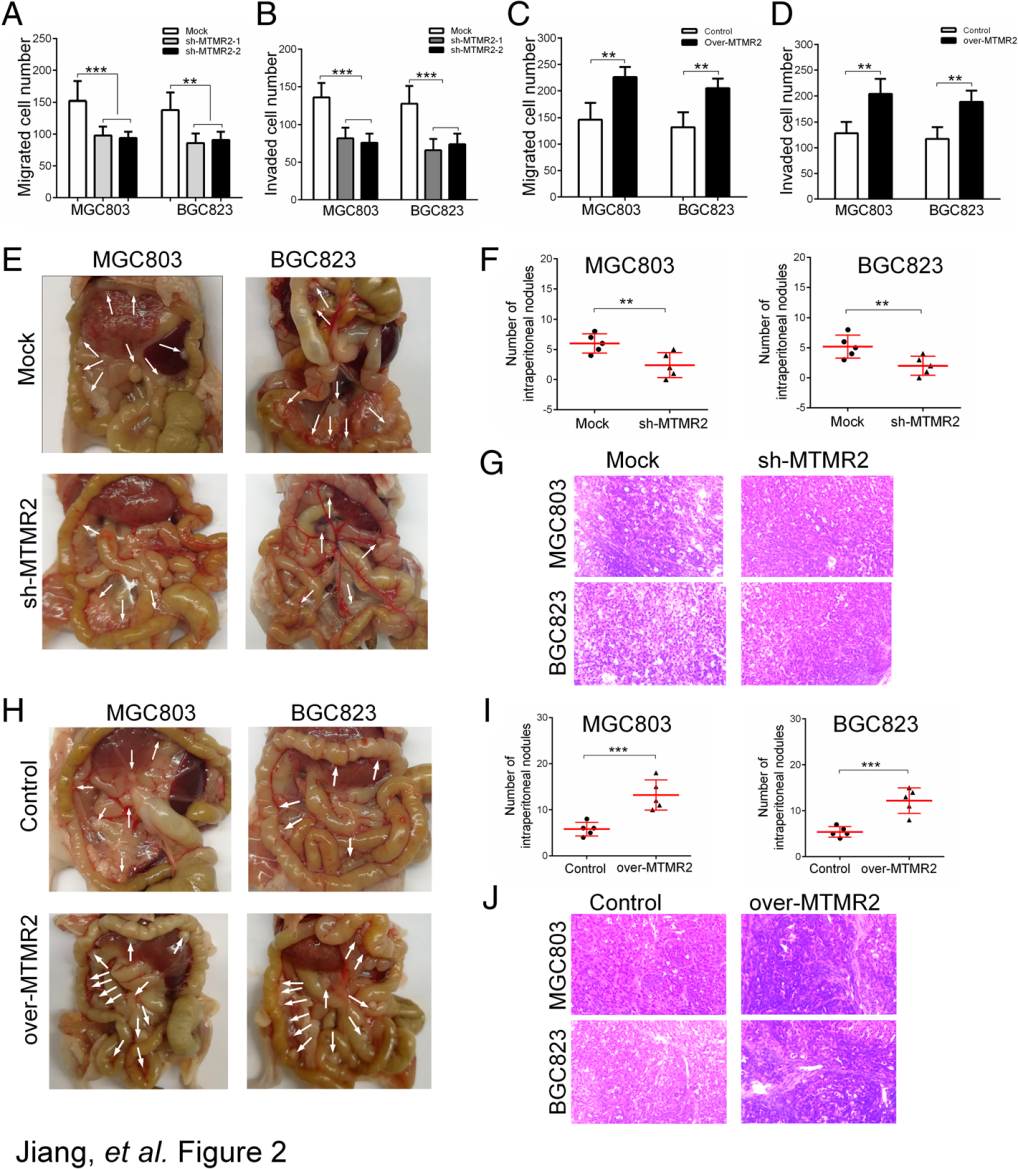
MTMR2 promotes the invasion and metastasis of GC cells. **a** Quantification of transwell migration assay showed that the migratory ability of sh-MTMR2 MGC803 and BGC823 cells was markedly reduced compared to mock cells, **, *P* < 0.01, ***, *P* < 0.001. **b** Quantification of transwell invasion assay showed that the invasive ability of sh-MTMR2 MGC803 and BGC823 cells was markedly reduced compared to mock cells. **c** Quantification of transwell migration assay showed that the migratory ability of over-MTMR2 MGC803 and BGC823 cells was markedly enhanced compared to control cells. **d** Quantification of transwell invasion assay showed that the invasive ability of over-MTMR2 MGC803 and BGC823 cells was markedly enhanced compared to control cells. **e** Representative images of intraperitoneal metastasis assay showed that the metastatic nodules derived from sh-MTMR2 MGC803 and BGC823 cells were fewer than those derived from the mock cells. **f** Quantification of intraperitoneal metastatic nodes formed by sh-MTMR2 MGC803 and BGC823 cells and mock cells. **g** H&E staining confirmed the origination of GC for intraperitoneal metastatic nodules. **h**, **i** Increased metastatic capability of over-MTMR2 MGC803 and BGC823 cells compared with control cells. **j** H&E staining confirmed the origination of GC for intraperitoneal metastatic nodules

Our clinical findings revealed that MTMR2 expression was correlated with depth of invasion and lymph node metastasis in GC specimens, implying that MTMR2 might involve in the invasion and metastasis of GC. To address this issue, the applicable cell models were established. The expression levels of MTMR2 in five GC cell lines (SGC7901, BGC823, MKN-45, AGC and MGC803) were higher than that in a gastric epithelial cell line (GES-1), with the highest expression in MGC803 and BGC823 cells (Additional file 1: Figure S5A and B). Hence, stably knocking-down and over-expressing of MTMR2 in MGC803 and BGC823 cells was conducted (Additional file 1: Figure S6A-D). With the MTMR2 gene-manipulated GC cells, we first examined the role of MTMR2 in migration and invasion of GC cells in vitro. MTMR2 knockdown significantly decreased the migratory and invasive capabilities in MGC803 and BGC823 cells (Fig. 2a and b, Additional file 1: Figures S7A-D, S8A and B). While overexpressing MTMR2 significantly increased the migratory and invasive capabilities in MGC803 and BGC823 cells (Fig. 2c and d, Additional file 1: Figures S7E-H, S8C and D). Then an intraperitoneal metastasis model was used to investigate the effect of MTMR2 on metastasis ability in nude mice. Silencing MTMR2 markedly reduced the metastatic capability (Fig. 2e-g), whereas MTMR2 overexpression markedly enhanced the metastatic capability in MGC803 and BGC823 GC cells (Fig. 2h-k). All metastatic nodules formed by GC cells in mice were confirmed as human GC origin by H&E staining (Fig. 2g-k). These results suggest that MTMR2 is an important player in the invasion and metastasis of GC cells.

### MTMR2 induces epithelial-mesenchymal-transition in GC cells

**Fig. 3:**
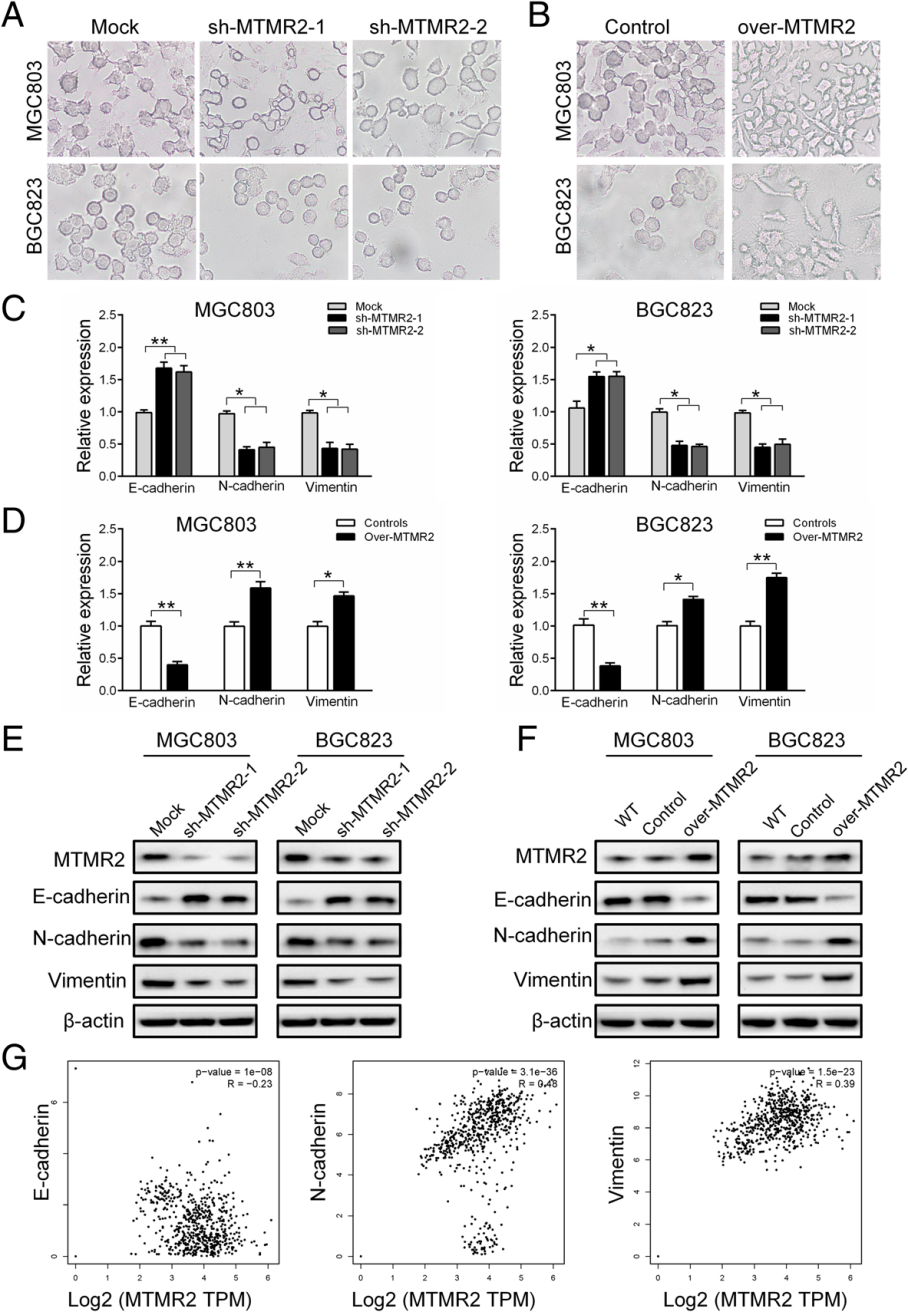
MTMR2 induces epithelial-mesenchymal transition in GC cells. **a**, **b** Representative images of morphology showed that silencing MTMR2 resulted in reduced cellular protrusions (**a**), while overexpressing MTMR2 resulted in not only increased cellular protrusions but also more spindle-like shape in partial cells in MGC803 and BGC823 GC cells (**b**). **c** qRT-PCR analyses showed that MTMR2 knockdown upregulated E-cadherin and downregulated N-cadherin and vimentin at mRNA level in MGC803 and BGC823 cells, ^*^, *P* < 0.05; ^**^, *P* < 0.001. **d** qRT-PCR analyses showed that MTMR2 overexpression downregulated E-cadherin and upregulated N-cadherin and vimentin at mRNA level in MGC803 cells and BGC823 cells, *, P < 0.05; **, *P* < 0.01. **e** Western blot analyses showed that MTMR2 knockdown upregulated E-cadherin and downregulated N-cadherin and vimentin at protein level in MGC803 and BGC823 cells. **f** Western blot analyses showed that MTMR2 overexpressing downregulated E-cadherin and upregulated N-cadherin and vimentin in MGC803 and BGC823 cells. **g** MTMR2 expression was correlated with the expression of E-cadherin, N-cadherin and vimentin in GC samples from TCGA dataset (*P* < 0.001 for all)

Epithelial-mesenchymal transition (EMT) has been well recognized as a key event in driving invasion and metastasis of GC cells [18]. We explored whether EMT process was involved in MTMR2-promoted the invasion and metastasis in GC cells. Silencing MTMR2, the cellular protrusions were markedly reduced in MGC803 and BGC823 GC cells (Fig. 3a). In contrast, overexpressing MTMR2 resulted in not only increased cellular protrusions but also more spindle-like shape in partial GC cells (Fig. 3b). Compared with the mock cells, MTMR2-knockdown MGC803 and BGC823 GC cells expressed higher level of E-cadherin and lower level of N-cadherin and vimentin at mRNA and protein levels (Fig. 3c, e). The reverse results at mRNA and protein level were observed by overexpression of MTMR2 in those cells (Fig. 3d, f). The analysis of TCGA data showed that the MTMR2 expression level was negatively correlated with E-cadherin and positively correlated with N-cadherin and vimentin (*P* < 0.05 for all) (Fig. 3g). Taken together, these results suggest that MTMR2 enhances the invasion and metastasis mainly by inducing EMT in GC cells.

### IFNγ/STAT1 signaling pathway is involved in MTMR2-induced EMT in GC cells

**Fig. 4:**
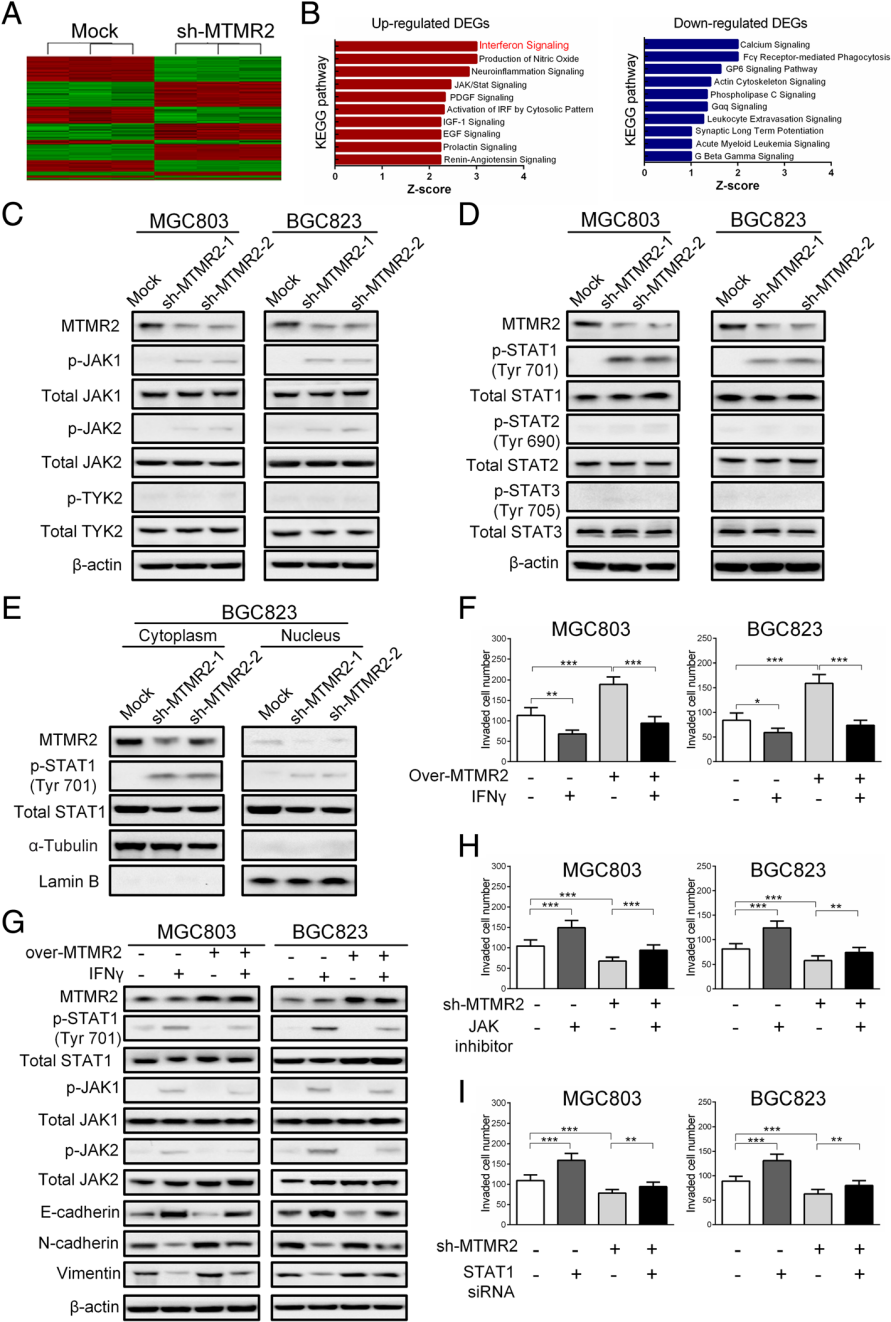
IFNγ/STAT1 signaling pathway is involved in MTMR2-induced EMT in GC cells. **a** Microarray heatmap showed differentially expressed genes in sh-MTMR2 BGC823 and mock cells. The color key for the normalized expression data was shown at the top of the microarray heatmap (green means downregulated genes and red represents upregulated genes. **b** Kyoto Encyclopedia of Genes and Genomes (KEGG) pathway enrichment analysis of the most differentially expressed genes between the sh-MTMR2 BGC823 and mock cells (fold change ≥1.5). All signaling pathways were sorted according to the Z score. **c** MTMR2 knockdown enhanced the phosphorylation of JAK1/2, but did not affected the phosphorylation of TYK2. **d** MTMR2 knockdown enhanced the phosphorylation of STAT1, but did not affected the phosphorylation of STAT2 and STAT3. **e** MTMR2 knockdown not only upregulated the level of pSTAT1, but also enhanced the nuclear accumulation of pSTAT1. **f** IFNγ treatment (50 ng/mL, 48 h) decreased invasion capability of MGC803 and BGC823 cells, which was attenuated by MTMR2-overexpression. **g** IFNγ treatment (50 ng/mL, 48 h) enhanced the levels of pJAK1/2 and pSTAT1, accompanying with upregulated E-cadherin and downregulated N-cadherin and vimentin. And these effects of IFNγ treatment were reversed by MTMR2 overexpression. **h**, **i** Both JAK inhibitor (15 ng/mL, 48 h) (**h**) and STAT1 siRNA (40 nmol/L, 48 h) (**i**) enhanced the invasive capability in control MGC803 and BGC823 cells and reversed the invasive capability reduced by MTMR2 knockdown in sh-MTMR2 MGC803 and BGC823 cells

To reveal the signaling pathways that involved in MTMR2-inducing EMT, a gene expression profiling was performed in sh-MTMR2 BGC823 and mock cells (Fig. 4a). After knockdown of MTMR2, a total of 695 differentially expressed genes were identified (Fold Changes > 1.5) in BGC823 GC cells. Among these genes, 357 were up-regulated and 338 were down-regulated. Consistent with our findings in in vitro studies, Ingenuity® Pathway Analysis (IPA®) showed that “migration of tumor cells”, “migration of cancer cells”, “cell movement of tumor cells” and “invasion of cells” ranked the top of “Diseases or Functions Annotation” related to MTMR2 depletion (Additional file 1: Table S6). KEGG pathway enrichment analyses indicated that interferon (IFN) signaling pathway was most affected by MTMR2 disruption (Fig. 4b). Previous studies have demonstrated that all IFNs signal through the Janus activated kinase (JAK) /signal transducer and activator of transcription (STAT) pathway, where engagement of the receptors induces sequential activation of JAK or tyrosine kinase 2 (TYK2) and STATs, resulting in transcriptional activation of interferon-stimulated genes [19]. Therefore, we first evaluated the affection of MTMR2 knockdown on the activation of JAKs and TYK2. Depletion of MTMR2 significantly upregulated the phosphorylation JAK1/2, but did not affect the phosphorylation of TYK2 both in MGC803 and BGC823 cells (Fig. 4c), implying that IFNγ signaling was involved. We then assessed the effect of MTMR2 knockdown on the activation of STATs. Depletion of MTMR2 significantly elevated the levels of phospho-STAT1 (pSTAT1) Tyr 701, but less affected the levels of phospho-STAT2 (pSTAT2) Tyr 690 and phospho-STAT3 (pSTAT3) Tyr705 or pSTAT3 Ser727 as well as total STAT2 and STAT3(Fig. 4c). Moreover, silencing of MTMR2 markedly increased the nuclear accumulation of pSTAT1 (Fig. 4d). Further IPA® and gene regulatory network analysis also identified that STAT1 and its target genes were the top molecules regulated by MTMR2 (Additional file 1: Figure S9). These results strongly suggested that IFNγ/STAT1 pathway of IFN signaling was involved in MTMR2-enhanced invasion and metastasis of GC cells. Hence, we stimulated GC cells with IFNγ (50 ng/mL), and found that invasive capability in control GC cells was markedly reduced and over-MTMR2-enhanced invasive capability in MTMR2-overexpressing cells was significantly attenuated (Fig. 4e, Additional file 1: Figure S12A). Because our results have suggested that MTMR2 enhanced invasion and metastasis mainly by inducing EMT in GC cells, we further assessed the affection of IFNγ/STAT1 pathway on EMT. IFNγ treatment enhanced the phosphorylation of STAT1, JAK1/2, accompanying with upregulated E-cadherin and downregulated N-cadherin and vimentin, which were reversed by MTMR2 overexpression (Fig. 4f). Treatment with JAK inhibitor I, an inhibitor of JAK1/2, not only enhanced the invasive capability in control GC cells but also reversed sh-MTMR2-reduced invasive capability in sh-MTMR2 cells (Fig. 4g, Additional file 1: Figure S10B). Similar results were observed after treatment with STAT1 siRNA (Fig. 4h, Additional file 1: Figure S10C). Thus, MTMR2 acts as an important inactivation factor of IFNγ/STAT1 pathway to induce EMT in GC cells.

### MTMR2 inactivating IFNγ/STAT1 to upregulate ZEB1

**Fig. 5:**
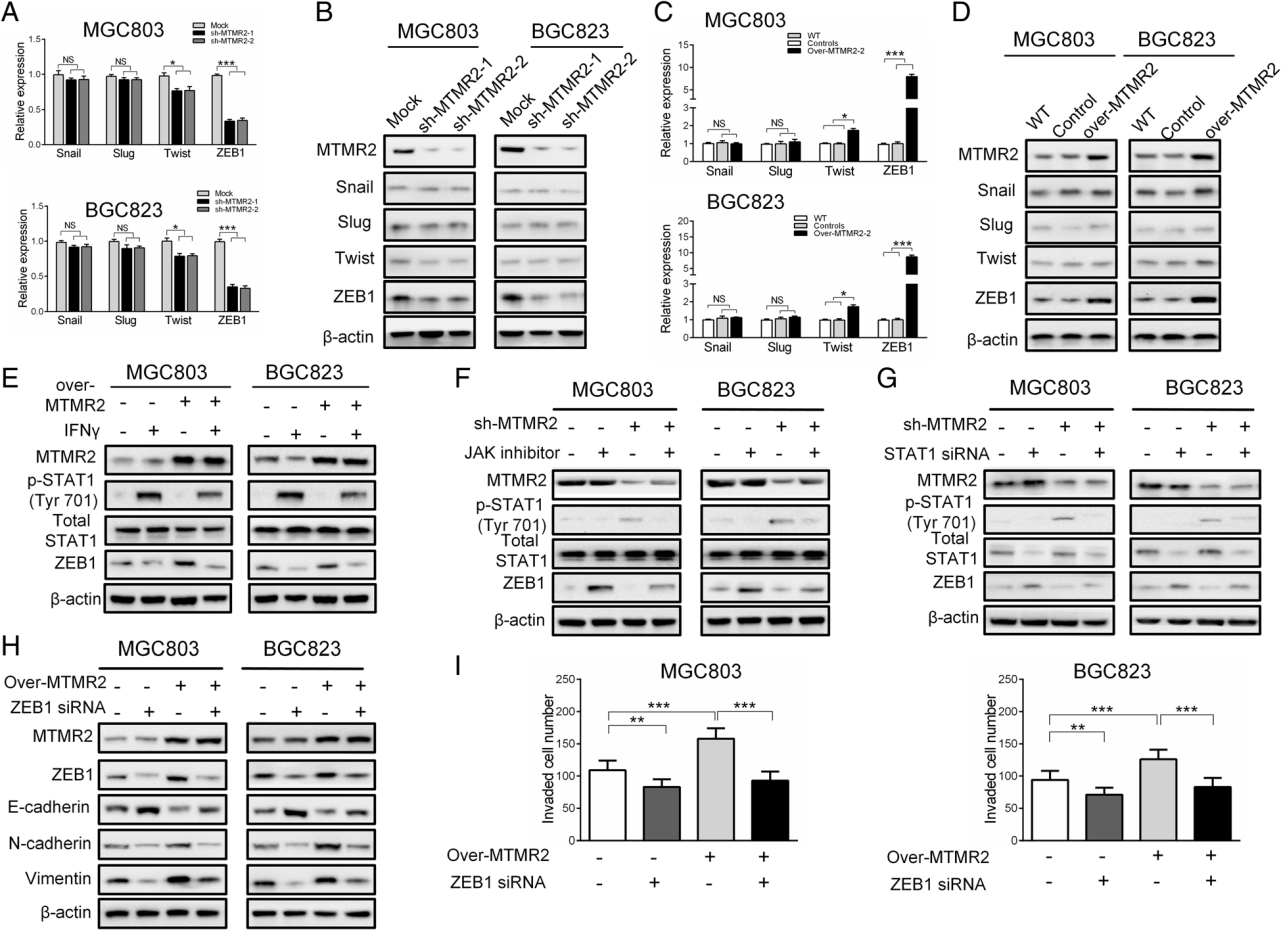
ZEB1 mediates MTMR2-induced EMT by inactivating IFNγ/STAT1 in GC cells. **a**, **b** qRT-PCR and Western blot showed that MTMR2 knockdown significantly decreased ZEB1 expression at both mRNA (**a**) and protein (**b**) levels in sh-MTMR2 MGC803 and BGC823 cells, while the expression of Twist, Snail and Slug were not or less affected. **c**, **d** qRT-PCR and Western blot showed that overexpressing MTMR2 markedly increased ZEB1 expression at both mRNA (**c**) and protein (**d**) levels in over-MTMR2 MGC803 and BGC823 cells, but less affected the expression of Twist, Snail and Slug. **e** IFNγ treatment (50 ng/mL, 48 h) increased phosphorylation of STAT1 and downregulated ZEB1 in control MGC803 and BGC823 cells, and attenuated the upregulation of MTMR2 overexpression on ZEB1 in over-MTMR2 cells. **f**, **g** Both JAK inhibitor (**f**) and STAT1 siRNA (**g**) decreased phosphorylation of STAT1 and upregulated ZEB1 in mock MGC803 and BGC823 cells, and reversed the downregulation of MTMR2 knockdown on ZEB1 in sh-MTMR2 MGC803 and BGC823 cells. **h** Depletion of ZEB1 significantly upregulated E-cadherin and downregulated N-cadherin and vimentin in sh-MTMR2 and mock MGC803 and BGC823 cells. **i** Depletion of ZEB1 significantly decreased the invasive ability in sh-MTMR2 and mock MGC803 and BGC823 cells, **, *P* < 0.01, ***, *P* < 0.001

Since IFNγ/STAT1 signaling has been reported to be involved in regulation of EMT-related transcription factors [20–23], we therefore examined the effect of MTMR2 knockdown on the expression of EMT-related transcription factors. Silencing of MTMR2 in MGC803 and BGC823 cells markedly downregulated ZEB1 and slightly downregulated Twist, but did not affect Snail and Slug at both mRNA and protein levels (Fig. 5a and b). The opposite results were observed upon overexpression of MTMR2 (Fig. 5c and d), suggesting that ZEB1 was the main EMT-related transcription factor involving in MTMR2-induced EMT in GC cells. Treatment with IFNγ increased phosphorylation of STAT1 and downregulated ZEB1 in control cells, simultaneously reversed MTMR2 overexpression-inhibited phosphorylation of STAT1 and -increased expression of ZEB1 in over-MTMR2 cells (Fig. 5e). Since transcriptional factor STAT1 was phosphorylated by the protein kinases JAKs, we therefore treated over-MTMR2 and control GC cells with JAK inhibitor. Treatment with JAK inhibitor abolished the phosphorylation of STAT1 and restored the expression of ZEB1 induced by MTMR2 knockdown in sh-MTMR2 GC cells (Fig. 5f). Transfection with STAT1-targeting siRNA significantly reduced both total and phosphorylated STAT1 accompanying upregulation of ZEB1 in mock cells, and attenuated the effect of MTMR2-knockdown on the upregulation of both total and phosphorylated STAT1 as well as downregulation of ZEB1 (Fig. 5g). Depletion of ZEB1 significantly upregulated E-cadherin and downregulated N-cadherin and vimentin in control cells, and reversed the effect of MTMR2-overexpression on the downregulation of E-cadherin and upregulation of N-cadherin and vimentin in over-MTMR2 GC cells (Fig. 5h). Depletion of ZEB1 also significantly decreased the invasive ability of control cells, and attenuated the effect of MTMR2-overexpression on the promotion of invasive ability in sh-MTMR2 GC cells (Fig. 5i, Additional file 1: Figure S11). These findings indicate that MTMR2 upregulates ZEB1 to induce EMT by inactivating IFNγ/STAT1 pathway in GC cells.

### IRF1 mediates the inhibition of IFNγ/STAT1 on ZEB1

**Fig. 6:**
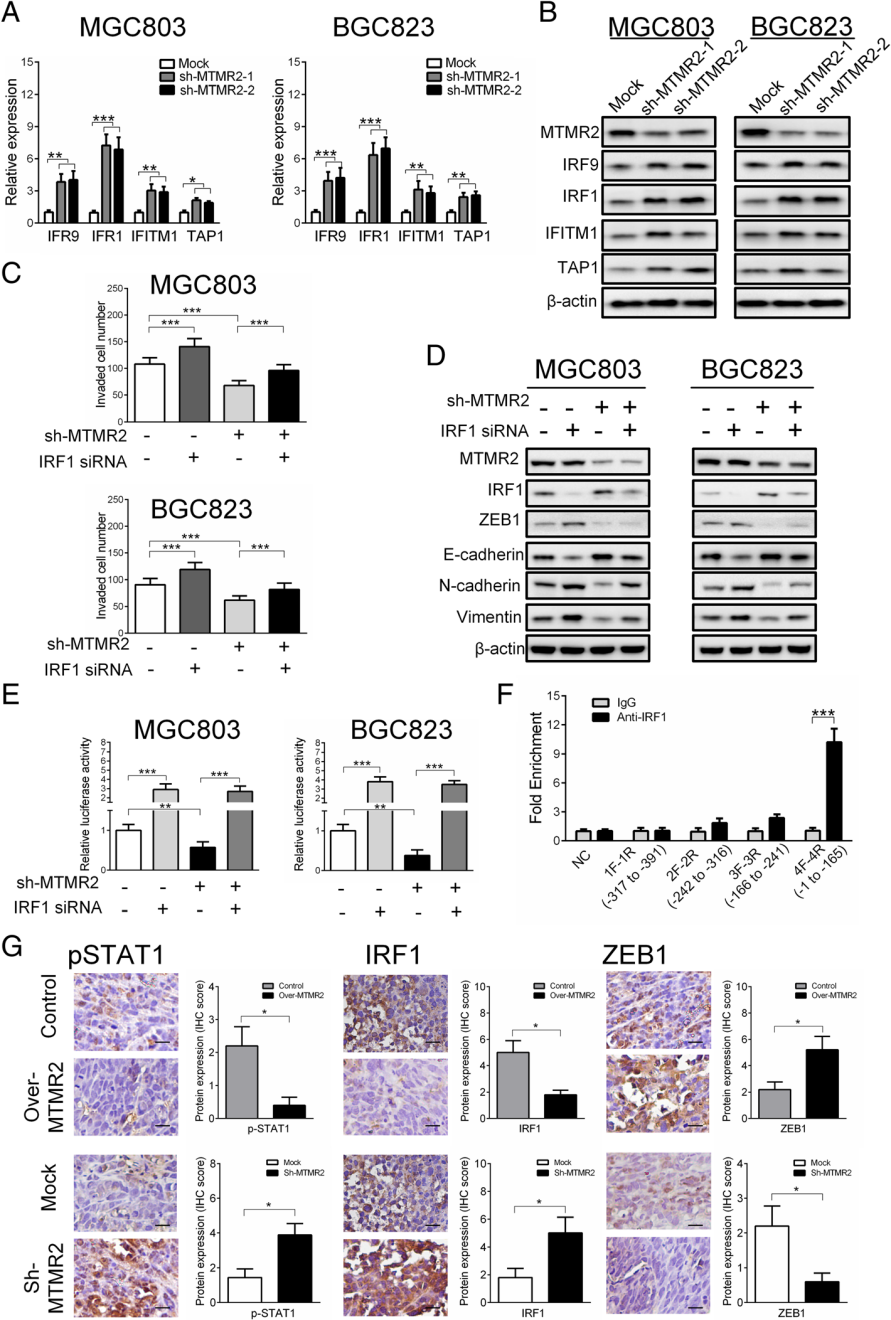
IRF1 mediates the inhibition of IFNγ/STAT1 on ZEB1 in GC cells. **a**, **b** Silencing of MTMR2 upregulated the expression of STAT1 targeted genes at both mRNA (**a**) and protein (**b**) levels, including IRF9, IRF1, IFITM1 and TAP1, with the most significant upregulation of IRF1 in MGC803 and BGC823 cells. **c** Silencing IRF1 significantly increased the invasion capacity in mock cells, and abrogated the inhibitory effect of MTMR2-knockdown on the invasion in sh-MTMR2 MGC803 and BGC823 cells. **d** Silencing IRF1 with siRNA (40 nmol/L) downregulated E-cadherin and upregulated N-cadherin and vimentin in mock MGC803 and BGC823 cells, and attenuated MTMR2 knockdown-induced upregulation of E-cadherin and downregulation of N-cadherin and vimentin in sh-MTMR2 MGC803 and BGC823 cells. **e** IRF1 siRNA (40 nmol/L) significantly increased ZEB1 promoter activity in mock MGC803 and BGC823 cells and sh-MTMR2 MGC803 and BGC823 cells, **, *P* < 0.01, ***, *P* < 0.001. **f** ChIP assay showed a candidate binding region (4F to 4R, − 1 to − 165) of IRF1 in ZEB1 promoter. **g** Representative images of IHC staining of pSTAT1, IRF1 and ZEB1 in intraperitoneal metastasis tumor tissue sections (original magnification, × 400) and the IHC scores of pSTAT1, IRF1 and ZEB1 protein expression, * *P* < 0.05

Finally, we asked how STAT1 regulates ZEB1 in GC cells. It has been reported that STAT1 exerts function by targeting several genes, including IRF9, IRF1, IFITM1 and TAP1 [24–26]. We therefore examined the change of these molecules after silencing of MTMR2. We found that MTMR2 knockdown upregulated the mRNA and protein expression of IRF9, IRF1, IFITM1 and TAP1, with the most significant upregulation of IRF1, implying that IRF1 may be the main mediator in process of STAT1 regulating ZEB1 (Fig. 6a, b). We then evaluated the effect of IRF1 on the invasion capacities of GC cells*.* Silencing IRF1 significantly increased the invasion capacity in mock cells, and abrogated the inhibitory effect of MTMR2-knockdown on the invasion in sh-MTMR2 cells (Fig. 6c, Additional file 1: Figure S12). Knockdown of IRF1 expression also resulted in down-regulation of the E-cadherin and up-regulation of N-cadherin and vimentin in mock cells, and attenuated MTMR2 knockdown-induced upregulation of E-cadherin and downregulation of N-cadherin and vimentin in sh-MTMR2 cells (Fig. 6d). To identify the pattern of IRF1 regulating ZEB1, transcriptional activity of ZEB1 promoter was measured by using luciferase reporter assays. Treatment with IRF1 siRNA significantly increased ZEB1 promoter activity in mock cells, and reversed MTMR2 knockdown-induced suppression of ZEB1 promoter activity in sh-MTMR2 cells (Fig. 6e), implying that IRF1 directly inhibits the transcription of ZEB1 gene in GC cells. To confirm the interaction between IRF1 and the promoter of ZEB1, a ChIP assay was performed with 4 pairs of primers covering − 391 to − 1 bp of the ZEB1 promoter. The results showed that the region of − 165 to -1 bp in ZEB1 promoter was a potential binding region for IRF1, in which there is a predictive binding site of − 81 to − 61 (Fig. 6f). These findings suggest that STAT1 regulating ZEB1 is mainly mediated by IRF1.

In addition, we examined the levels of pSTAT1, IRF1 and ZEB1 expression in intraperitoneal metastasis models established with MTMR2-knockdown or -overexpression MGC803 cells by IHC to confirm the involvement of IFNγ/STAT1/IRF1/ZEB1 pathway in MTMR2-mediated metastasis. Compared with the metastatic foci derived from control cells, the metastatic foci derived from MTMR2 overexpression cells exhibited significantly downregulated of pSTAT1 (*P* < 0.05) and IRF1 (P < 0.05) and upregulated ZEB1 (*P* < 0.05), while sh-MTMR2 achieved the opposite results (Fig. 6g). These results confirm the importance of IFNγ/STAT1/IRF1/ZEB1 pathway in MTMR2-mediated metastasis proven by in vitro experiments.

## Discussion

It is well-established that invasion-metastasis cascade drives the progression of cancer cells, and eventually leads to significantly poor outcome of the patients [18, 27, 28]. Despite the tremendous efforts have been made, the molecule mechanisms underlying the invasion and metastasis of GC remain largely unclear. In this study, we demonstrated that MTMR2 highly expressed in GC tissues and its expression levels were significantly correlated with invasion depth and lymph node metastasis, as well as the outcome of the patients. To the best of our knowledge, this is the first study to reveal the role and clinical relevance of MTMR2 in GC.

EMT has been demonstrated to play an important role in triggering the invasion and metastasis during tumor progression, by which epithelial origin cancer cells, including GC cells, lose their epithelial characteristics and acquire more migratory mesenchymal properties [18, 27, 28]. In this study, we demonstrated that MTMR2-promoting invasion and metastasis is closely associated with induction of EMT.

We performed a gene expression profiling to explore the potential signaling pathways that involved in MTMR2-inducing EMT and consequent invasion and metastasis in GC cells and uncovered the IFN signaling was most affected signaling after MTMR2 knockdown. Actually, there are three major types of IFN, i.e. type I (IFN-α, β, ε, κ, and ω), type II (Interferon-γ, IFNγ) and type III (IFN-λ1–4) [29]. Hence, it should be clarified which IFN signaling is involved in the process of MTMR2-induced EMT. Though all three types of IFNs signal through the JAK/STAT pathway, typically, type I and III IFN signaling activates TYK2 and JAK1, resulting in STAT1 heterodimerization, whereas type II IFN signaling activates JAK1 and JAK2 to induce STAT1 homodimerization [19]. Our results that depletion of MTMR2 significantly upregulated the phosphorylation JAK1/2, but did not affect the phosphorylation of TYK2 strongly suggested that MTMR2 induced EMT mainly through regulating type II IFN signaling.

IFNγ is associated with an antitumor role via extrinsic or tumor cell intrinsic mechanism [30]. Regarding the extrinsic antitumor property, IFNγ plays a pivotal function in stimulating antitumor immunity and promoting tumor recognition and elimination [31–33]. Regarding the tumor cell intrinsic mechanism of antitumor, many studies demonstrated that IFNγ signaling exerts antitumor role mainly involved in anti-proliferative and pro-apoptotic processes. Chin et al. reported that IFNγ inhibit the growth of tumor cells by activating STAT1 to upregulating cyclin-dependent kinase (CDK) inhibitor 1 [34]. Fulda et al. reported that IFNγ promotes tumor cell apoptosis by activating STAT1/IRF1 pathway to upregulate the expression of caspase-8 [35]. Wee et al. reported that EZH2 directly repressed the expression of interferon-gamma receptor 1 (IFNGR1) to inhibiting activation of IFN-JAK-STAT1 tumor-suppressor signaling and apoptosis in a MYC-dependent manner in a subset of metastatic prostate cancers [36]. In recent years, IFNγ/STAT1 pathway has been linked to metastatic progression of cancers. Varikuti et al. showed that STAT1 is an important suppressor of primary breast tumor growth and metastasis [37]. Several studies also suggested that IFNγ/STAT1 signal pathway plays a key role in inhibition of EMT in several cancers, including lung cancers [20], gallbladder cancer [21], hepatocellular carcinoma [22]. In this study, we demonstrated that MTMR2 inactivates IFNγ/STAT1 signaling resulted in induction of EMT and enhanced invasion and metastasis in GC cells. However, recent studies suggested that IFNγ/STAT1 signaling also exerts protumor effects. Kunita et al. [38] report that IFNγ/STAT1 stimulated the expression of cell surface protein podoplanin (PDPN), a potent inducer of cancer cell invasion, in the invasive front of squamous cell carcinomas (SCCs) of the cervix in patients and in the transgenic human papillomavirus/estrogen mouse model of cervical cancer. Lo et al. reported that IFNγ induced the transcription of interferon-induced tetratricopeptide repeat 5 (IFIT5) via the JAK-STAT signaling pathway to degrade the precursors of suppressive miRNAs of EMT, resulting in EMT in prostate cancer cells [39]. The opposite tumoricidal and protumor effects of IFNγ/STAT1 signaling may be attributed to the difference of the tumor-specific context, the magnitude of the signal, and the microenvironmental cues [30].

Activated JAKs act as the signal transducers downstream of IFNγ receptors and are characterized by phosphorylation [40, 41]. Our data suggested that MTMR2 is involved in the negative regulation of JAK1/2 phosphorylation in GC cells. Many studies have demonstrated that dephosphorization of phosphorylated JAKs catalyzed by protein tyrosine phosphatases (PTPs), such as SHP1, SHP2, CD45, PTPRD, PTPRT and so on, is one key regulatory module [42]. Although MTMR2 is initially described as a protein tyrosine/dual specificity phosphatase based on the primary sequence, but no any substrate of phosphoprotein has been identified so far [43]. Numerous studies have now shown that MTMR2 uses phosphoinositide lipids (PIs), rather than phosphoproteins, as physiological substrates, involving in converting lipid second messengers PI(3)P and PI(3, 5)P2 into PI and PI(5) P [44–46]. Therefore, the mechanisms underlying MTMR2 inhibiting the phosphorylation of JAKs remained to be further elucidated.

IRF1 is an important downstream molecule of JAKs-STAT1 signaling, and one of the major primary response genes induced by STAT1 signaling [30]. Upon activated by STAT1, IRF1 functions as a transcription activator of interferon-stimulated response elements (ISRE), leading to the transcription of a large number of secondary response genes [47, 48]. IRF1 mainly functions as a tumor suppressor protein, low-expressing in several human cancers including breast cancer [49], endometrial carcinoma [50], leukemia [51], hepatocellular carcinoma [52] and cholangiocarcinoma [53]. Moreover, IRF1 has associated with inhibition of EMT, migration and invasion of some cancer cells [54–56]. Lin YH et al. reported that IRF1 regulates the expression of EMT-related transcription factors Snail, Slug and Twist [23]. However, we found that IRF1 suppresses the EMT and invasion by directly inhibiting the expression of ZEB1 at transcriptional level in GC cells.

## Conclusions

In summary, we demonstrate that MTMR2 promotes EMT, invasion and metastasis by inactivating the IFNγ/JAK/STAT1/IRF1 signaling, a canonical pathway for IFNγ, to provoke ZEB1 expression in GC cells. MTMR2 is high expressed in GC tissues and the expression level is closely correlated with clinicopathological features and outcome of the patients, suggesting that MTMR2 may serve as a prognostic biomarker and a potentially therapeutic target in GC.

## Additional file

Additional file 1: **Table S1.** Patient characteristics. **Table S2.** Sequences of MTMR2 knockdown shRNAs and a scramble (mock) used in this study. **Table S3.** The siRNA sequences used in this study. **Table S4.** Sequences of primers used for qRT-PCR in this study. **Table S5.** Primary and secondary antibodies for IHC and western blot. **Table S6.** 15 Top diseases or functions annotation after MTMR2-knockdown according to an IPA. **Table S7.** Sequences of primers for chromatin-immunoprecipitation (ChIP). **Figure S1.** Identification of optimal cutoff value. **Figure S2.** MTMR2 expression was associated with clinicopathological features in GC. **Figure S3.** MTMR2 expression correlates with prognosis of GC patients stratified by MTMR2 expression. **Figure S4.** MTMR2 expression was an independent prognostic factor for GC patients. **Figure S5.** The expression of MTMR2 in normal gastric cell line and GC cell lines. **Figure S6.** The efficiency of silencing and over-expressing MTMR2 in GC cells. **Figure S7.** The results of wound-healing assay for MTMR2 knock-down or overexpression in GC cells. **Figure S8.** Representative images of matrigel-transwell invasion assay for MTMR2 knock-down or overexpression in GC cell. **Figure S9.** Interferon signaling retrieved from ingenuity pathway analysis (IPA). **Figure S10.** Representative images of matrigel transwell invasion of GC cells. **Figure S11.** Representative images of matrigel transwell invasion assay for sh-MTMR2 GC cells treated with or without ZEB1 siRNA (50 nmol/L). **Figure S12.** Representative images of matrigel transwell invasion assay for sh-MTMR2 GC cells treated with or without IRF1 siRNA (50 nmol/L). (DOC 25394 kb)

## Abbreviations

CHIP: Chromatin immunoprecipitation
DFS: Disease free survival
EMT: Epithelial-mesenchymal transition
FBS: Fetal bovine serum
GC: Gastric cancer
HR: Hazard ratio
IFN: Interferon
IHC: Immunohistochemistry
JAK: Janus activated kinase
MTMR2: Myotubularin-related protein 2
OS: Overall survival
PBS: Phosphate buffer saline
PTPs: Protein tyrosine phosphatases
qRT-PCR: Quantitative real-time PCR
STAT: Signal transducer and activator of transcription
TNM: Tumor-node-metastasis

## References

[1] Bray F, Ferlay J, Soerjomataram I, Siegel RL, Torre LA, Jemal A (2018). Global cancer statistics 2018: GLOBOCAN estimates of incidence and mortality worldwide for 36 cancers in 185 countries. CA Cancer J Clin.

[2] Chen W, Zheng R, Baade PD, Zhang S, Zeng H, Bray F (2016). Cancer statistics in China, 2015. CA Cancer J Clin.

[3] Wadhwa R, Song S, Lee JS, Yao Y, Wei Q, Ajani JA (2013). Gastric cancer-molecular and clinical dimensions. Nat Rev Clin Oncol.

[4] Van Cutsem E, Sagaert X, Topal B, Haustermans K, Prenen H (2016). Gastric cancer. Lancet..

[5] Mruk DD, Cheng CY (2011). The myotubularin family of lipid phosphatases in disease and in spermatogenesis. Biochem J.

[6] Hnia K, Vaccari I, Bolino A, Laporte J (2012). Myotubularin phosphoinositide phosphatases: cellular functions and disease pathophysiology. Trends Mol Med.

[7] Chojnowski A, Ravise N, Bachelin C, Depienne C, Ruberg M, Brugg B (2007). Silencing of the Charcot-Marie-Tooth associated MTMR2 gene decreases proliferation and enhances cell death in primary cultures of Schwann cells. Neurobiol Dis.

[8] Bolino A, Muglia M, Conforti FL, LeGuern E, Salih MA, Georgiou DM (2000). Charcot-Marie-tooth type 4B is caused by mutations in the gene encoding myotubularin-related protein-2. Nat Genet.

[9] Bolino A, Bolis A, Previtali SC, Dina G, Bussini S, Dati G (2004). Disruption of Mtmr2 produces CMT4B1-like neuropathy with myelin outfolding and impaired spermatogenesis. J Cell Biol.

[10] Brenner DR, Amos CI, Brhane Y, Timofeeva MN, Caporaso N, Wang Y (2015). Identification of lung cancer histology-specific variants applying Bayesian framework variant prioritization approaches within the TRICL and ILCCO consortia. Carcinogenesis..

[11] Izykowska K, Przybylski GK, Gand C, Braun FC, Grabarczyk P, Kuss AW (2017). Genetic rearrangements result in altered gene expression and novel fusion transcripts in Sezary syndrome. Oncotarget..

[12] Amin MB, Edeg S, Byrd DR (2016). AJCC cancer staging Manua [M]l.

[13] Liu JY, Jiang L, Liu JJ, He T, Cui YH, Qian F (2018). AEBP1 promotes epithelial-mesenchymal transition of gastric cancer cells by activating the NF-kappaB pathway and predicts poor outcome of the patients. Sci Rep.

[14] McCarty KS, Miller LS, Cox EB, Konrath J, McCarty KS (1985). Estrogen receptor analyses. Correlation of biochemical and immunohistochemical methods using monoclonal antireceptor antibodies. Arch Pathol Lab Med.

[15] Camp RL, Dolled-Filhart M, Rimm DL (2004). X-tile: a new bio-informatics tool for biomarker assessment and outcome-based cut-point optimization. Clin Cancer Res.

[16] Schmittgen TD, Livak KJ (2008). Analyzing real-time PCR data by the comparative C(T) method. Nat Protoc.

[17] Chandrashekar DS, Bashel B, Balasubramanya SAH, Creighton CJ, Ponce-Rodriguez I, Chakravarthi B (2017). UALCAN: a portal for facilitating tumor subgroup gene expression and survival analyses. Neoplasia..

[18] Nieto MA, Huang RY, Jackson RA, Thiery JP (2016). Emt: 2016. Cell..

[19] Chow KT, Gale M (2015). SnapShot: interferon signaling. Cell..

[20] Kachroo P, Lee MH, Ling Z, Baratelli F, Lee G, Srivastava MK (2013). IL-27 inhibits epithelial-mesenchymal transition and angiogenic factor production in a STAT1-dominant pathway in human non-small cell lung cancer. J Exp Clin Cancer Res.

[21] Shen H, Zhan M, Zhang Y, Huang S, Xu S, Huang X (2018). PLZF inhibits proliferation and metastasis of gallbladder cancer by regulating IFIT2. Cell Death Dis.

[22] Chen Yongyan, Hao Xiaolei, Sun Rui, Wei Haiming, Tian Zhigang (2019). Natural Killer Cell–Derived Interferon‐Gamma Promotes Hepatocellular Carcinoma Through the Epithelial Cell Adhesion Molecule–Epithelial‐to‐Mesenchymal Transition Axis in Hepatitis B Virus Transgenic Mice. Hepatology.

[23] Lin YH, Wu MH, Liao CJ, Huang YH, Chi HC, Wu SM (2015). Repression of microRNA-130b by thyroid hormone enhances cell motility. J Hepatol.

[24] Park J, Kim J, Park B, Yang KM, Sun EJ, Tognon CE (2018). Novel identification of STAT1 as a crucial mediator of ETV6-NTRK3-induced tumorigenesis. Oncogene..

[25] Zhang Y, Liu Z (2017). STAT1 in cancer: friend or foe?. Discov Med.

[26] Ivashkiv Lionel B. (2018). IFNγ: signalling, epigenetics and roles in immunity, metabolism, disease and cancer immunotherapy. Nature Reviews Immunology.

[27] Jean Paul T (2002). Epithelial-mesenchymal transitions in tumour progression. Nat Rev Cancer.

[28] RA W (2009). The basics of epithelial-mesenchymal transition. J Clin Invest.

[29] Parker BS, Rautela J, Hertzog PJ (2016). Antitumour actions of interferons: implications for cancer therapy. Nat Rev Cancer.

[30] Castro F, Cardoso AP, Gonça lves RM, Serre K, Oliveira MJ. Interferon-gamma at the crossroads of tumor immune surveillance or evasion. Front Immunol. 2018;9:847. 10.3389/fimmu.2018.00847 PMC5945880 29780381

[31] Shankaran V, Ikeda H, Bruce AT, White JM, Swanson PE, Old LJ (2001). IFNgamma and lymphocytes prevent primary tumour development and shape tumour immunogenicity. Nature..

[32] Lu W, Yan W, Zhiyu S, Jiahui C, Xianjun Q (2015). Deficiency of interferon-gamma or its receptor promotes colorectal cancer development. J Interf Cytokine Res.

[33] Kaplan DH, Shankaran V, Dighe AS, Stockert E, Aguet M, Old LJ (1998). Demonstration of an interferon gamma-dependent tumor surveillance system in immunocompetent mice. Proc Natl Acad Sci U S A.

[34] Rodriguez A, Jung EJ, Yin Q, Cayrol C, Flemington EK (2001). Role of c-myc regulation in Zta-mediated induction of the cyclin-dependent kinase inhibitors p21 and p27 and cell growth arrest. Virology..

[35] Simone F, Klaus-Michael D (2002). IFNgamma sensitizes for apoptosis by upregulating caspase-8 expression through the Stat1 pathway. Oncogene..

[36] Wee ZN, Li Z, Lee PL, Lee ST, Lim YP, Yu Q (2014). EZH2-mediated inactivation of IFN-γ-JAK-STAT1 signaling is an effective therapeutic target in MYC-driven prostate cancer. Cell Rep.

[37] Varikuti S, Oghumu S, Elbaz M, Volpedo G, Ahirwar DK, Alarcon PC (2017). STAT1 gene deficient mice develop accelerated breast cancer growth and metastasis which is reduced by IL-17 blockade. Oncoimmunology..

[38] Kunita A, Baeriswyl V, Meda C, Cabuy E, Takeshita K, Giraudo E (2018). Inflammatory cytokines induce podoplanin expression at the tumor invasive front. Am J Pathol.

[39] Lo UG, Pong RC, Yang D, Gandee L, Hernandez E, Dang A, et al. IFN-γ-induced IFIT5 promotes epithelial-to-mesenchymal transition in prostate cancer via microRNA processing. Cancer Res. 2018. 10.1158/0008-5472.CAN-18-2207 30504123

[40] Wilks AF, Harpur AG, Kurban RR, Ralph SJ, Zurcher G, Ziemiecki A (1991). Two novel protein-tyrosine kinases, each with a second phosphotransferase-related catalytic domain, define a new class of protein kinase. Mol Cell Biol.

[41] Firmbach-Kraft I, Byers M, Shows T, Dalla-Favera R, Krolewski JJ (1990). tyk2, prototype of a novel class of non-receptor tyrosine kinase genes. Oncogene..

[42] Babon JJ, Lucet IS, Murphy JM, Nicola NA, Varghese LN (2014). The molecular regulation of Janus kinase (JAK) activation. Biochem J.

[43] Robinson FL, Dixon JE (2006). Myotubularin phosphatases: policing 3-phosphoinositides. Trends in Cell Biol.

[44] Zhao R, Qi Y, Chen J, Zhao ZJ (2001). FYVE-DSP2, a FYVE domain-containing dual specificity protein phosphatase that dephosphorylates phosphotidylinositol 3-phosphate. Exp Cell Res.

[45] Taylor GS, Maehama T, Dixon JE (2000). Myotubularin, a protein tyrosine phosphatase mutated in myotubular myopathy, dephosphorylates the lipid second messenger, phosphatidylinositol 3-phosphate. Proc Natl Acad Sci U S A.

[46] Kim SA, Taylor GS, Torgersen KM, Dixon JE (2002). Myotubularin and MTMR2, phosphatidylinositol 3-phosphatases mutated in myotubular myopathy and type 4B Charcot-Marie-tooth disease. J Biol Chem.

[47] Kroger A, Dallugge A, Kirchhoff S, Hauser H (2003). IRF-1 reverts the transformed phenotype of oncogenically transformed cells in vitro and in vivo. Oncogene..

[48] Schwartz-Roberts JL, Cook KL, Chen C, Shajahan-Haq AN, Axelrod M, Warri A (2015). Interferon regulatory factor-1 signaling regulates the switch between autophagy and apoptosis to determine breast cancer cell fate. Cancer Res.

[49] Stang MT, Armstrong MJ, Watson GA, Sung KY, Liu Y, Ren B (2007). Interferon regulatory factor-1-induced apoptosis mediated by a ligand-independent fas-associated death domain pathway in breast cancer cells. Oncogene..

[50] Giatromanolaki A, Koukourakis MI, Ritis K, Mimidis K, Sivridis E (2004). Interferon regulatory factor-1 (IRF-1) suppression and derepression during endometrial tumorigenesis and cancer progression. Cytokine..

[51] Dimitrios T, Matthaios S, Konstantinos A, Christina V, Sofia K, George T (2015). Low expression of interferon regulatory factor-1 and identification of novel exons skipping in patients with chronic myeloid leukaemia. Br J Haematol.

[52] Tzoanopoulos D, Speletas M, Arvanitidis K, Veiopoulou C, Kyriaki S, Thyphronitis G (2002). Low expression of interferon regulatory factor-1 and identification of novel exons skipping in patients with chronic myeloid leukaemia. Br J Haematol.

[53] Wan P, Zhang J, Du Q, Geller DA (2018). The clinical significance and biological function of interferon regulatory factor 1 in cholangiocarcinoma. Biomed Pharmacother.

[54] Cheng L, Geng L, Dai B, Zheng T, Fu J, Qiao L (2018). Repression of let-7a cluster prevents adhesion of colorectal cancer cells by enforcing a mesenchymal phenotype in presence of liver inflammation. Cell Death Dis.

[55] Li Y, Chang Z, Yanxia L, Min H, Zuoyang Z, Zheying Z (2015). IFN-γ-mediated IRF1/miR-29b feedback loop suppresses colorectal cancer cell growth and metastasis by repressing IGF1. Cancer Lett.

[56] Wan P, Chi X, Du Q, Luo J, Cui X, Dong K (2018). miR-383 promotes cholangiocarcinoma cell proliferation, migration, and invasion through targeting IRF1. J Cell Biochem.

