# Supplementary material for: MTMR2 promotes invasion and metastasis of gastric cancer via inactivating IFNγ/STAT1 signaling
Source: J Exp Clin Cancer Res. 2019 May 21;38:206. doi: 10.1186/s13046-019-1186-z (PMC6528261; doi:10.1186/s13046-019-1186-z)
Supplement: Supplementary file 1 — Table S1. Patient characteristics. Table S2. Sequences of MTMR2 knockdown shRNAs and a scramble (mock) used in this study. Table S3. The siRNA sequences used in this study. Table S4. Sequences of primers used for qRT-PCR in this study. Table S5. Primary and secondary antibodies for IHC and western blot. Table S6. 15 Top diseases or functions annotation after MTMR2-knockdown according to an IPA. Table S7. Sequences of primers for chromatin-immunoprecipitation (ChIP). Figure S1. Identification of optimal cutoff value. Figure S2. MTMR2 expression was associated with clinicopathological features in GC. Figure S3. MTMR2 expression correlates with prognosis of GC patients stratified by MTMR2 expression. Figure S4. MTMR2 expression was an independent prognostic factor for GC patients. Figure S5. The expression of MTMR2 in normal gastric cell line and GC cell lines. Figure S6. The efficiency of silencing and over-expressing MTMR2 in GC cells. Figure S7. The results of wound-healing assay for MTMR2 knock-down or overexpression in GC cells. Figure S8. Representative images of matrigel-transwell invasion assay for MTMR2 knock-down or overexpression in GC cell. Figure S9. Interferon signaling retrieved from ingenuity pathway analysis (IPA). Figure S10. Representative images of matrigel transwell invasion of GC cells. Figure S11. Representative images of matrigel transwell invasion assay for sh-MTMR2 GC cells treated with or without ZEB1 siRNA (50 nmol/L). Figure S12. Representative images of matrigel transwell invasion assay for sh-MTMR2 GC cells treated with or without IRF1 siRNA (50 nmol/L). (DOC 25394 kb) [file 13046_2019_1186_MOESM1_ESM.doc]

**MTMR2 promotes invasion and metastasis of gastric cancer via inactivating** **IFNγ/STAT1 signaling**

**Supplementary Tables**

| **Table S1.** Patient characteristics | | |
| --- | --- | --- |
| Prognostic variables | Number | Percent (%) |
| **Sex** |  |  |
| Male | 206 | (69.83) |
| Female | 89 | (30.17) |
| **Age** |  |  |
| <60 | 185 | (62.71) |
| ≥60 | 110 | (37.29) |
| **Tumor location** |  |  |
| proximal | 64 | (21.70) |
| middle | 67 | (22.71) |
| distal | 164 | (55.59) |
| **Histological grade** |  |  |
| G1 | 9 | (3.05) |
| G2 | 78 | (26.44) |
| G3 | 208 | (70.51) |
| **Tumor size** |  |  |
| <5 cm | 200 | (67.80) |
| ≥5 cm | 95 | (32.20) |
| **T stage** |  |  |
| T1 | 26 | (8.81) |
| T2 | 54 | (18.31) |
| T3 | 91 | (30.85) |
| T4 | 124 | (42.03) |
| **N stage** |  |  |
| N0 | 96 | (32.54) |
| N1 | 61 | (20.68) |
| N2 | 76 | (25.76) |
| N3 | 62 | (21.02) |
| **M stage** |  |  |
| M0 | 276 | (93.56) |
| M1 | 19 | (6.44) |
| **TNM stage** |  | (5.42) |
| I | 52 | (17.63) |
| II | 91 | (30.85) |
| III | 133 | (45.08) |
| IV | 19 | (6.44) |
| **MTMR2 expression** |  |  |
| Low | 148 | (50.17) |
| High | 147 | (49.83) |

| **Table S2.** Sequences of MTMR2 knockdown shRNAs and a scramble (mock) used in this study | | |
| --- | --- | --- |
| Gene | Sequence | |
| Sh-MTMR2-1 | Forward | 5′-CACCGCCAGACATGGGTGATGTACACGAA  TGTACATCACCCATGTCTGGC-3′ |
|  | Reverse | 5′-AAAAGCCAGACATGGGTGATGTACATTCG  TGTACATCACCCATGTCTGGC-3′ |
| Sh-MTMR2-2 | Forward | 5′-CACCGCTATGAGGAAATGACCTTTCCGAAGAAAGGTCATTTCCTCATAGC-3′ |
|  | Reverse | 5′-AAAAGCTATGAGGAAATGACCTTTCTTCGG  AAAGGTCATTTCCTCATAGC-3′ |
| Mock | Forward | 5′-CCGGTTACGCGTAGCGTAATACGCTCGAGC  GTATTACGCTACGCGTAATTTTTG-3′ |
|  | Reverse | 5′-AATTCAAAAATTACGCGTAGCGTAATACGCTCGAG CGTATTACGCTACGCGTAA-3′ |

| **Table S3.** The siRNA sequences used in this study | |
| --- | --- |
| Gene | Sequence |
| IRF1 | 5′-CCAGCGACCUGUACAACUU-3′ |
|  | 3′-AAGUUGUACAGGUCGCUGG-5′ |
| NC | 5′-UUCUCCGAACGUGUCACGU-3′ |
|  | 3′-ACGUGACACGUUCGGAGAA-5′ |
| STAT1 siRNA I | 5′-CGAGAGCUGUCUAGGUUAAC-3′ |
|  | 3′-GUUAACCUAGACAGCUCUCG-5′ |
| STAT1 siRNA II | 5′-GGGCAUCAUGCAUCUUACU-3′ |
|  | 3′-AGUAAGAUGCAUGAUGCCC-5′ |
| ZEB1 siRNA I | 5′-CCUAGUCAGCCACCUUUAATT-3′ |
|  | 3′-UUAAAGGUGGCUGACUAGGTT-5′ |
| ZEB1 siRNA II | 5′-GGAUCAACCACCAAUGGUUTT-3′ |
|  | 3′-AACCAUUGGUGGUUGAUCCTT-5′ |
| ZEB1 siRNA III | 5′-AACCAUUGGUGGUUGAUCCTT-3′ |
|  | 3′-UAAUAAUUCUCCCUGUUGCTT-5′ |

| **Table S4.** Sequences of primers used for qRT-PCR in this study | | |
| --- | --- | --- |
| Gene | Sequence | |
| MTMR2 | Forward | 5′-GAGGCCGTATCCCAGTTTTATC-3′ |
|  | Reverse | 5′-CTTTGCTTCGCTTTCCACTCA-3′ |
| E-cadherin | Forward | 5′-CTACAATGAGCTGCGTGTGG-3′ |
|  | Reverse | 5′-AGGTCCAGACGCAGGATGGC-3′ |
| N-cadherin | Forward | 5′-CAGTATCCGGTCCGATCTGC-3′ |
|  | Reverse | 5′-GTCCTGCTCACCACCACTAC-3′ |
| Vimentin | Forward | 5′-GACGCCATCAACACCGAGTT-3′ |
|  | Reverse | 5′-CTTTGTCGTTGGTTAGCTGGT-3′ |
| Snai1 | Forward | 5′-TCTGAGGCCAAGGATCTCCA-3′ |
|  | Reverse | 5′-TGGCTTCGGATGTGCATCTT-3′ |
| Slug | Forward | 5′-CATCTTTGGGGCGAGTGAGT-3′ |
|  | Reverse | 5′-ATGGCATGGGGGTCTGAAAG-3′ |
| Twist | Forward | 5′-TCTACCAGGTCCTCCAGAGC-3′ |
|  | Reverse | 5′-CTCCATCCTCCAGACCGAGA-3′ |
| ZEB1 | Forward | 5′-CAACTACGGTCAGCCCT-3′ |
|  | Reverse | 5′-GCGGTGTAGAATCAGAGTC-3′ |
| β-actin | Forward | 5′-GAATTCATGTTTGAGACCTTCAA-3′ |
|  | Reverse | 5′-CCGGATCCATCTCTTGCTCGAAGTCCA-3′ |
| IRF9 | Forward | 5′-GCCTGTAACACACTGCCTCT-3′ |
|  | Reverse | 5′-CTTGTAGGGCTCAGCAACAT-3′ |
| IRF1 | Forward | 5′-GACCCTGACCCGAAAACCT-3′ |
|  | Reverse | 5′-TGCTCCTGCTTCTTGCTT-3′ |
| IFITM1 | Forward | 5′-CGGCTCTGTGACAGTCTACC-3′ |
|  | Reverse | 5′-TGTATCTAGGGGCAGGACCA-3′ |
| TAP1 | Forward | 5′-GCAGTCAACTCCTGGACCACTA-3′ |
|  | Reverse | 5′-CAAGGTTCCCACTGCTTACAGC-3′ |

**Table S5. Primary and secondary antibodies for IHC and western blot**

| Antibody | Source | Dilution | Catalogue Number |
| --- | --- | --- | --- |
| MTMR2 | Santa Cruz | 1:500 | sc-365185 |
| Flag | abcam | 1:5000 | ab1257 |
| Phospho-STAT1 (Tyr701) | abcam | 1:500 | ab30645 |
| STAT1 | Santa Cruz | 1:500 | sc-464 |
| Phospho-JAK1(Tyr1034/1035) | CST | 1:1000 | 74129 |
| JAK1 | CST | 1:1000 | 50996 |
| Phospho-JAK2 (Tyr1007/1008) | CST | 1:1000 | 3771 |
| JAK2 | CST | 1:1000 | 4040 |
| Phospho-TYK2 (Tyr1054/1055) | CST | 1:1000 | 9321 |
| TYK2 | CST | 1:1000 | 9312 |
| Phospho-STAT2 (Tyr690) | CST | 1:2000 | 88410 |
| STAT2 | CST | 1:1000 | 72604 |
| Phospho-STAT3 (Tyr705) | CST | 1:2000 | 9145 |
| STAT3 | CST | 1:1000 | [12640](https://www.cst-c.com.cn/products/primary-antibodies/stat3-d3z2g-rabbit-mab/12640?site-search-type=Products&N=4294956287&Ntt=stat3&fromPage=plp) |
| β-actin | CST | 1:1000 | [4970](https://www.cst-c.com.cn/products/primary-antibodies/b-actin-13e5-rabbit-mab/4970?site-search-type=Products&N=4294956287&Ntt=Î²-actin&fromPage=plp) |
| α-Tubulin | CST | 1:1000 | 12351 |
| Lamin B | CST | 1:1000 | 15068 |
| IRF9 | CST | 1:1000 | 28492 |
| IRF1 | CST | 1:1000 | 8478 |
| IFITM1 | CST | 1:1000 | 13126 |
| TAP1 | CST | 1:1000 | 12341 |
| E-Cadherin | CST | 1:1000 | 14472 |
| N-Cadherin | Proteintech | 1:5000 | 22018-1-AP |
| Vimentin | CST | 1:1000 | 5741 |
| Snail | CST | 1:500 | 3879 |
| Slug | CST | 1:500 | 9585 |
| ZEB1 | CST | 1:500 | 3396 |
| Twist | CST | 1:500 | 46702 |
| Goat anti-mouse | Beyotime | 1:1000 | A0216 |
| Goat anti- rabbit | Beyotime | 1:1000 | A0208 |

Table S6. 15 Top diseases or functions annotation after MTMR2-knockdown according to an IPA

| **Diseases or Functions Annotation** | **Molecules** | **Activation z-score** |
| --- | --- | --- |
| Immune response of cells | 54 | 3.023 |
| Migration of tumor cells | 22 | 2.718 |
| Response of embryonic cell lines | 11 | 2.559 |
| Migration of cancer cells | 19 | 2.555 |
| Differentiation of epithelial tissue | 30 | 2.484 |
| Synthesis of nitric oxide | 25 | 2.445 |
| Large intestine neoplasm | 426 | 2.433 |
| Colorectal neoplasia | 270 | 2.433 |
| Antimicrobial response | 44 | 2.429 |
| Cell movement of tumor cells | 18 | 2.41 |
| Antiviral response | 40 | 2.405 |
| Invasion of cells | 76 | 2.396 |
| Response of kidney cell lines | 10 | 2.377 |
| Response of epithelial cell lines | 9 | 2.377 |
| Albuminuria | 10 | 2.375 |

| **Table S7: Sequences of primers for chromatin-immunoprecipitation (ChIP)** | | |
| --- | --- | --- |
| **Primer Sequences** | **Product length (bp)** | **Product sites** |
| 1F: TTTCTCCCTCCCCTCTGGGA  1R: AAAAATATAATTATGGATTG | 75 | -317 to -391 |
| 2F: TTCCTGTCTAGAAGCAGATA  2R: TCTTTAAAATGCAAGTGTTTA | 75 | -242 to -316 |
| 3F: AATATATTCGAGCCATCATT  3R: TCCACTCCTTGCTATAACAA | 76 | -166 to -241 |
| 4F: CGTCTGTTGATTATAAACGA  4R: AAAGCCACATCAGCAACAGCG | 165 | -1 to -165 |

**Supplementary Figures**


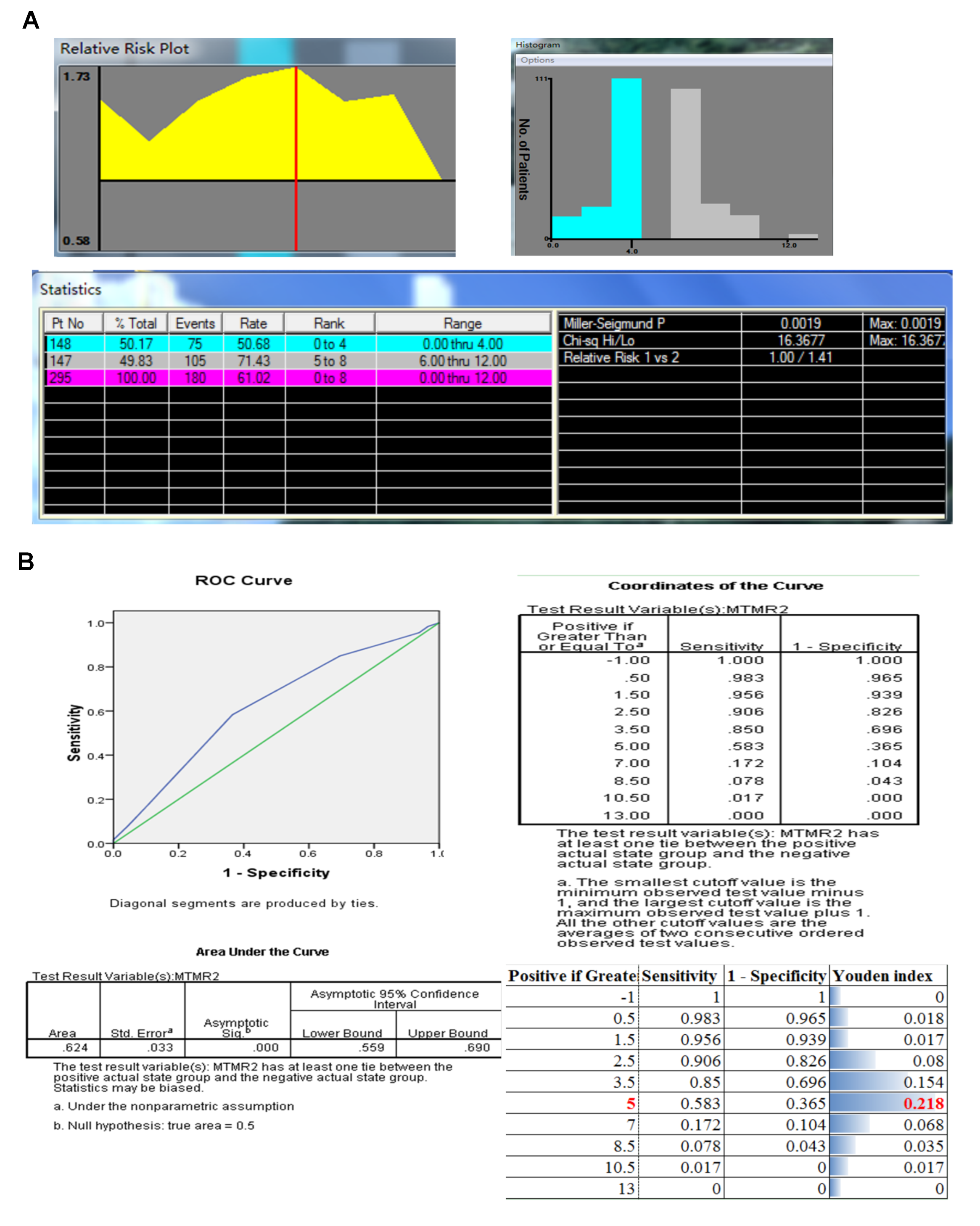


**Figure S1. Identification of optimal cutoff value. (A)** The relative risk plot of each IHC score generated by X-tile software, which showed that the optimal cutoff value was 4. **(B)** The ROC curve and Youden’s index analyzed by SPSS 19.0 software showed that the maximum value of Youden index was 0.218 when the IHC score was 5. Consequently, the optimal cutoff value is defined as 6.


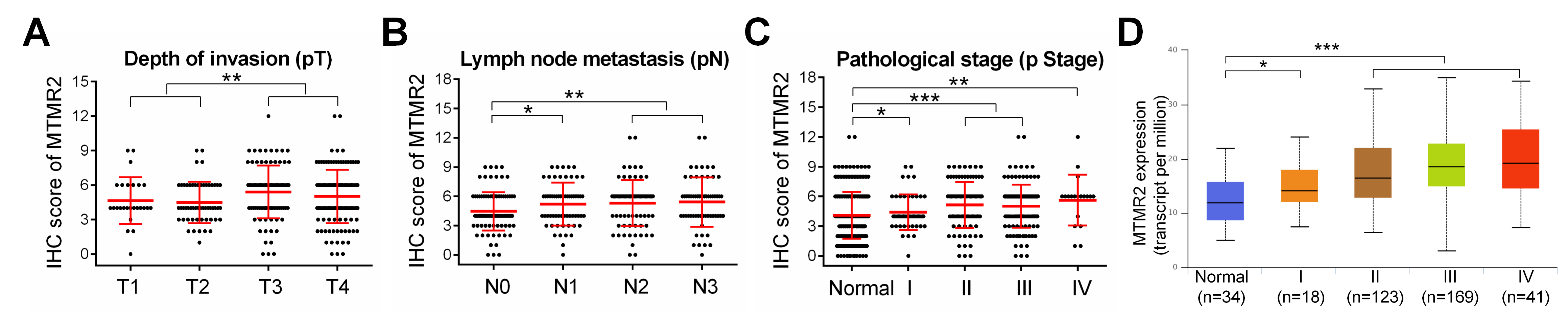


**Figure S2**. **MTMR2 expression was associated with clinicopathological features in GC. (A-C)** Scatter plot of MTMR2 IHC scores according to depth of invasion (A), lymph node metastasis (B), and pathological stage (C). **(D)** TCGA data showed that MTMR2 expression was associated with TNM stages analyzed by an online tool ualcan (http://ualcan.path.uab.edu/). *, p<0.05; ***, p<0.001.


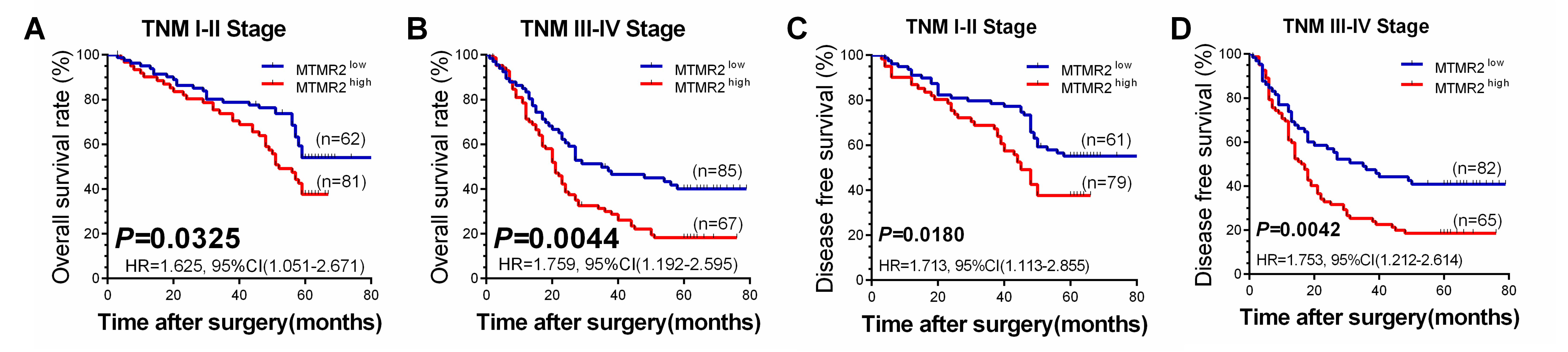


**Figure S3. MTMR2 expression correlates with prognosis of GC patients stratified by MTMR2 expression. (A, B)** Kaplan-Meier survival analysis for OS in stage I-II (A) and III-IV (B) GC patients from our cohort showed that high expression of MTMR2 was correlated with a lower overall survival rate. **(C, D)** Kaplan-Meier survival analysis for DFS in stage I-II (C) and III-IV (D) GC patients from our cohort showed that high expression of MTMR2 was correlated with a lower disease free survival rate.


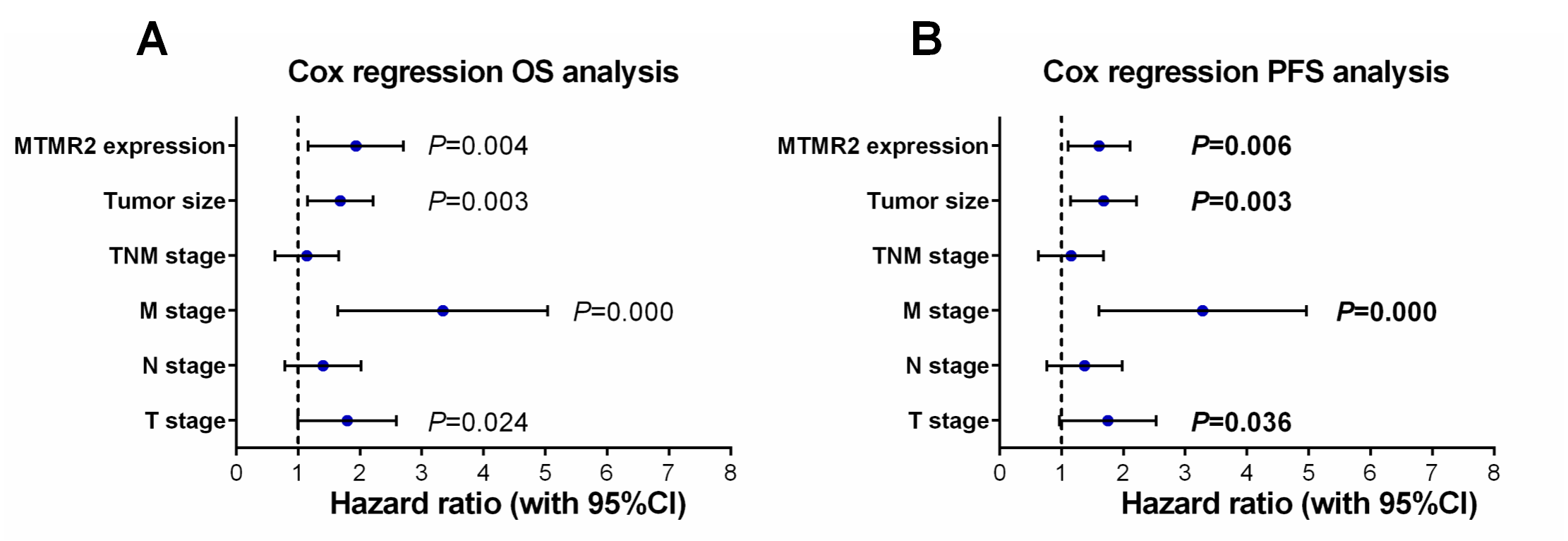


**Figure S4. MTMR2 expression was an independent prognostic factor for GC patients. (A, B)** Different risk factors including MTMR2 expression, tumor size, TNM stage, M stage, N stage and T stage were analyzed for their association with GC patients’ OS (A) and PFS (B) using Cox regression model. The hazard ratio (HR) and 95% confident interval (CI) are plotted for each risk factor.


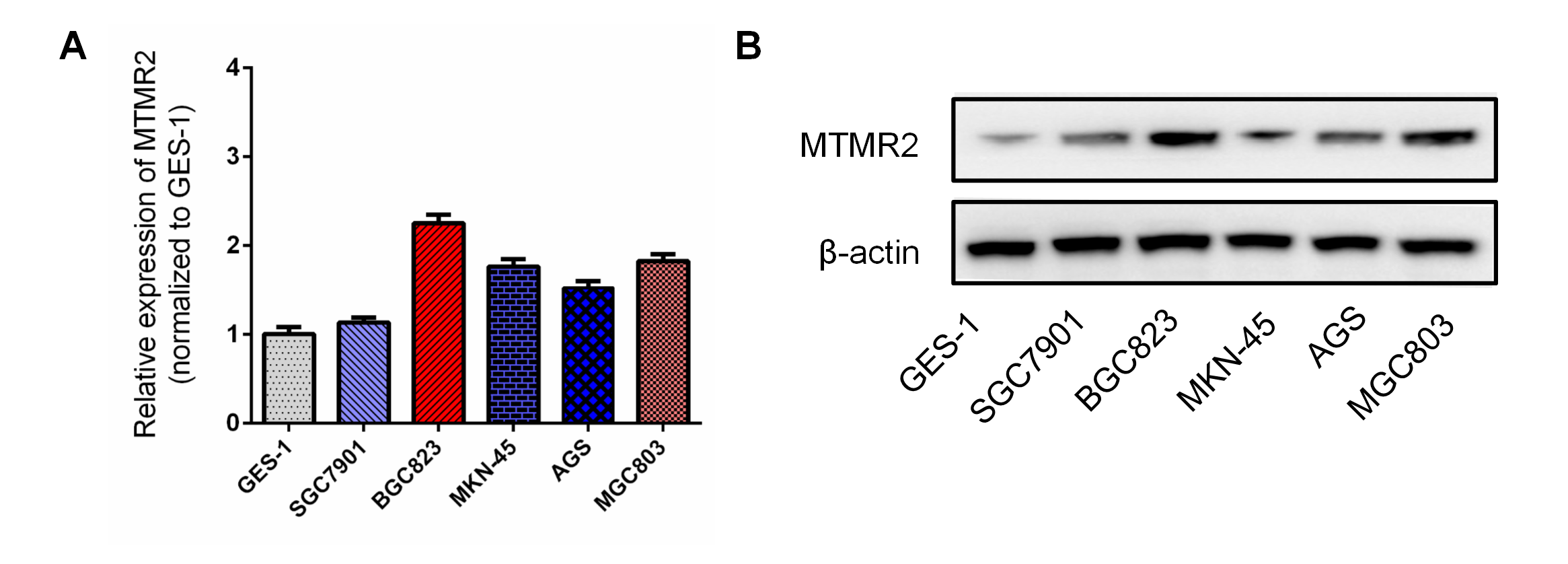


**Figure S5. The expression of MTMR2 in normal gastric cell line and GC cell lines. (A)** qRT-PCR analyses of MTMR2 expression at mRNA level in GES-1 and five GC cell lines (SGC7901, BGC823, MKN-45, AGC and MGC803); **(B)** Western blotting analyses of MTMR2 expression at protein level in GES-1 and five GC cell lines (SGC7901, BGC823, MKN-45, AGC and MGC803).


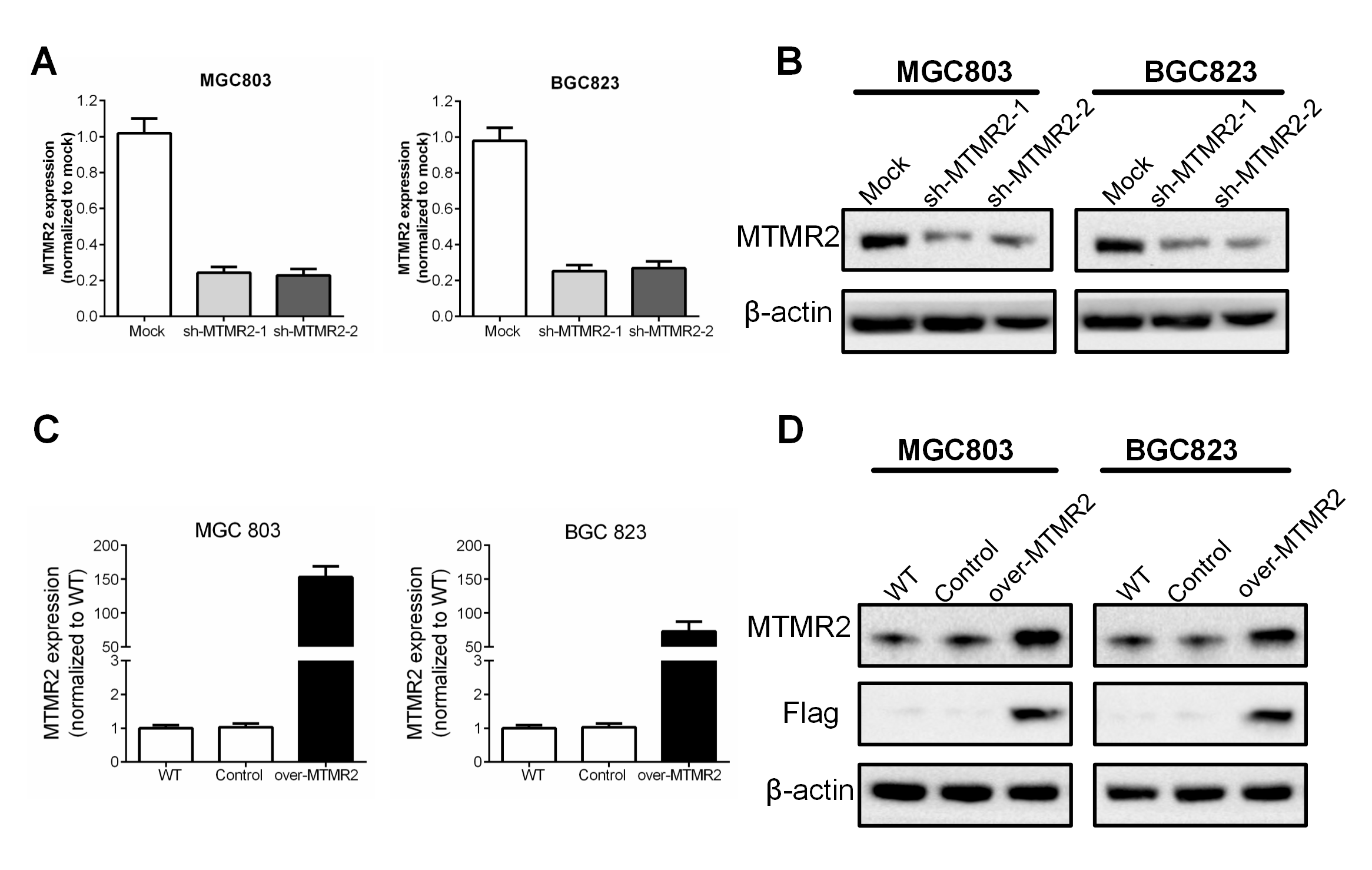


Figure S6. **The efficiency of silencing and over-expressing MTMR2 in GC cells. (**A, B**)** The efficiency of MTMR2 knockdown in MGC803 and BGC823 GC cells at mRNA (A) and protein (B) levels with sh-MTMR-1 showed the highest efficiency of MTMR2 silencing. **(**C, D**)** The efficiency of MTMR2 over-expression in MGC803 and BGC823 cells at mRNA (A) and protein (B) levels.


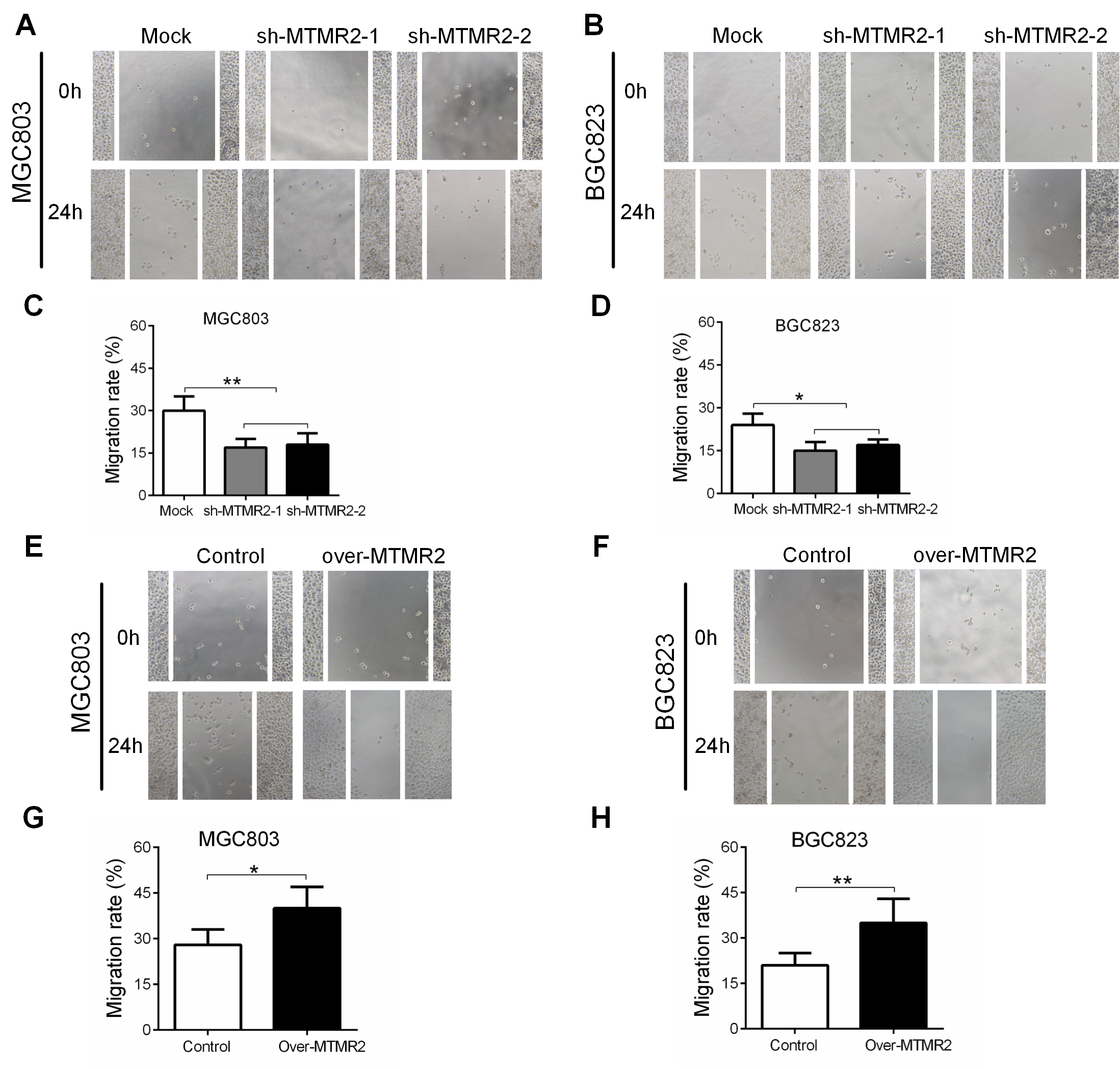


Figure S7. The results of wound-healing assay **for MTMR2 knock-down or overexpression in GC cells.** (A, B) Representative images of the wound-healing assay in MGC803 (A) and BGC823 (B) GC cells after MTMR2-knockdown; magnification: 200×. (C, D) Quantification of the wound healing assay results in MGC803 (C) and BGC823 (D) GC cells after MTMR2-knockdown. (E, F) Representative images of the wound-healing assay in MGC803 (E) and BGC823 (F) GC cells after MTMR2-overexpression; magnification: 200×. (G, H) Quantification of the wound healing assay results in MGC803 (G) and BGC823 (H) after MTMR2-verexpression.


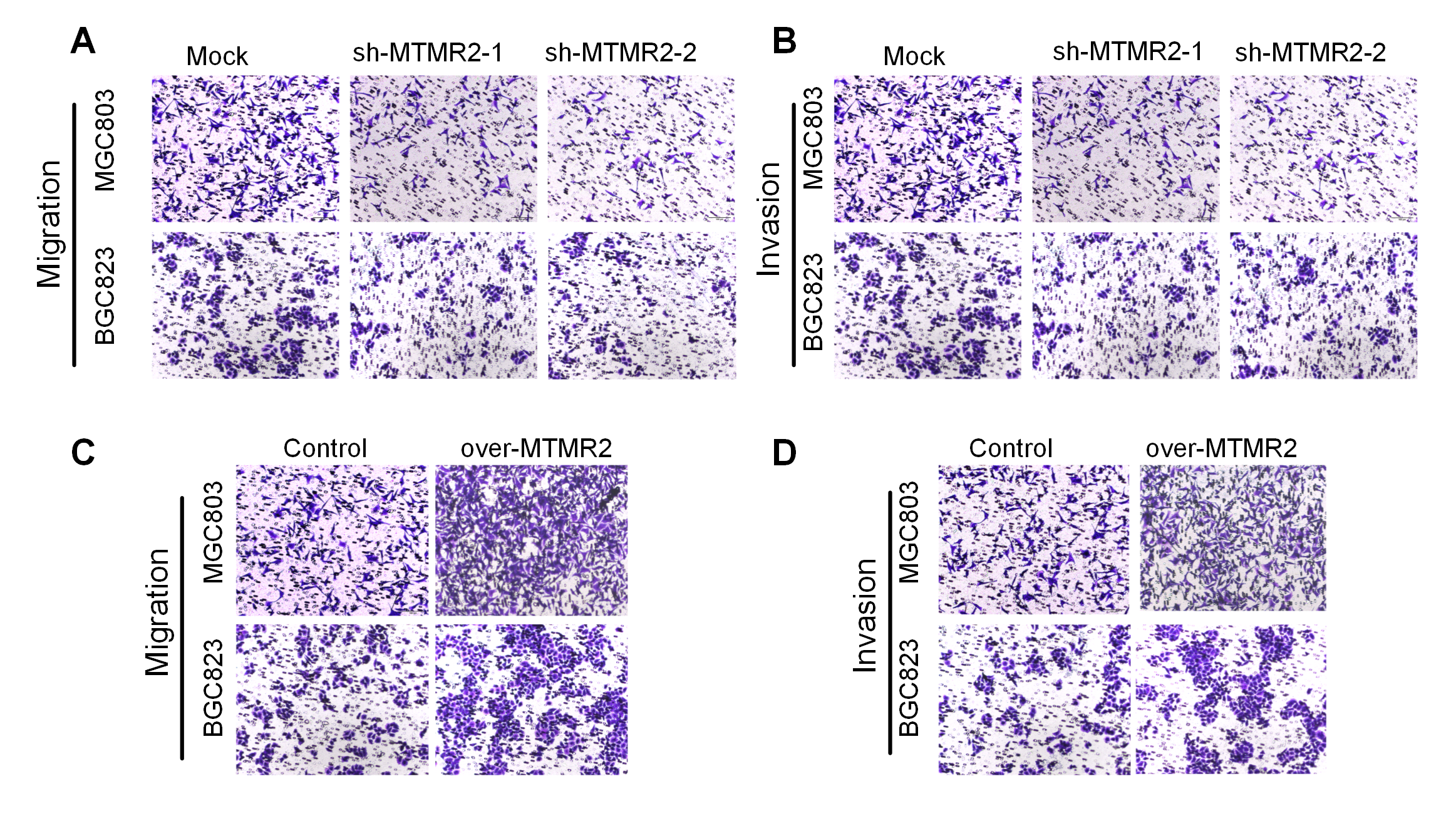


**Figure S8. Representative images of matrigel-transwell invasion assay for MTMR2 knock-down or overexpression in GC cell. (**A) Representative images of transwell migration assay in GC cells after MTMR2 knockdown, magnifcation: 200×. (B) Representative images of transwell invasion assay in GC cells after MTMR2 knockdown, magnifcation: 200×. (C) Representative images of transwell migration assay in GC cells after MTMR2-overexpression, magnifcation: 200×. (D) Representative images of transwell invasion assay in GC cells after MTMR2-overexpression, magnifcation: 200×.


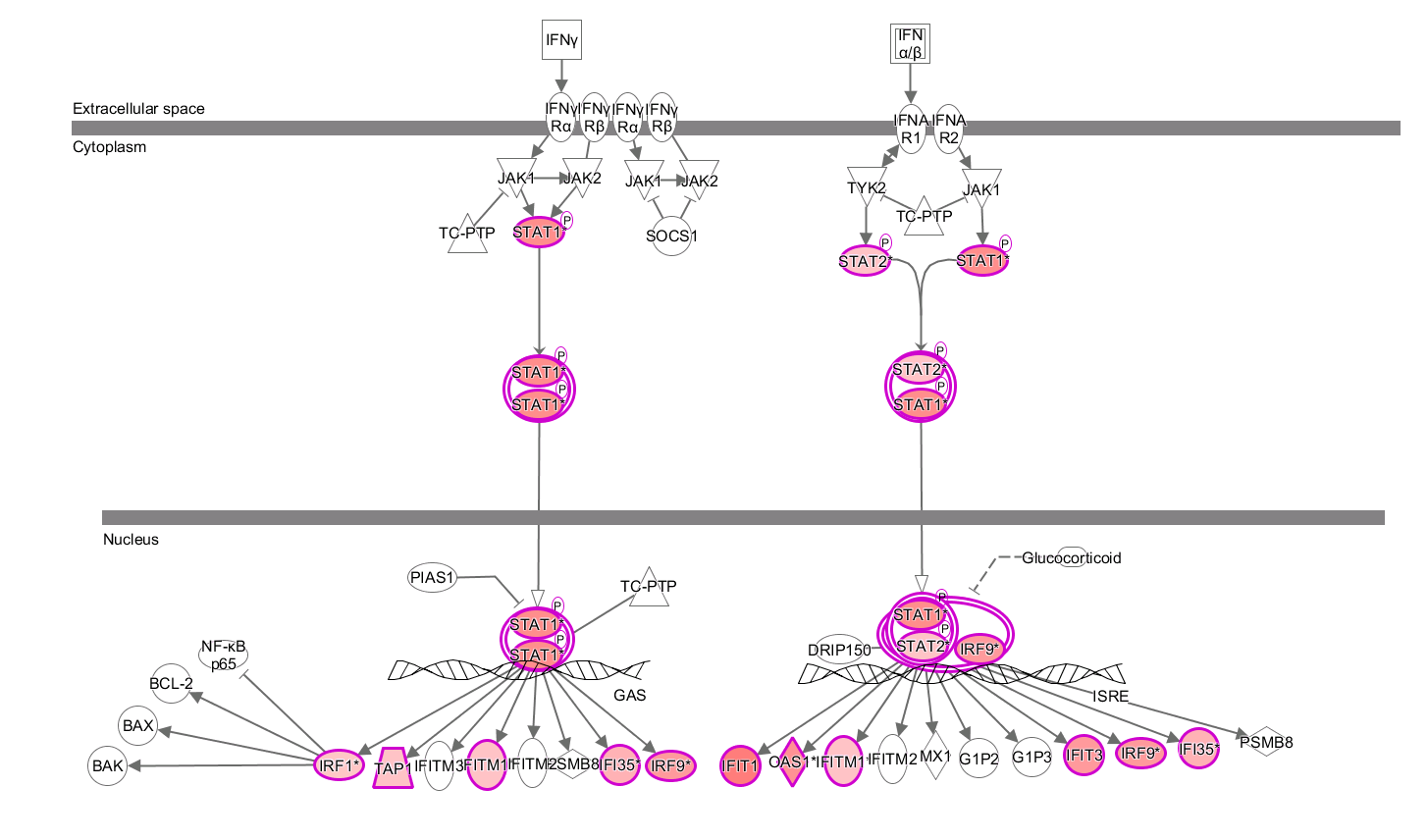


Figure S9. **Interferon signaling retrieved from ingenuity pathway analysis (IPA).** Red indicates up-regulated genes, and color intensity indicates the fold change.


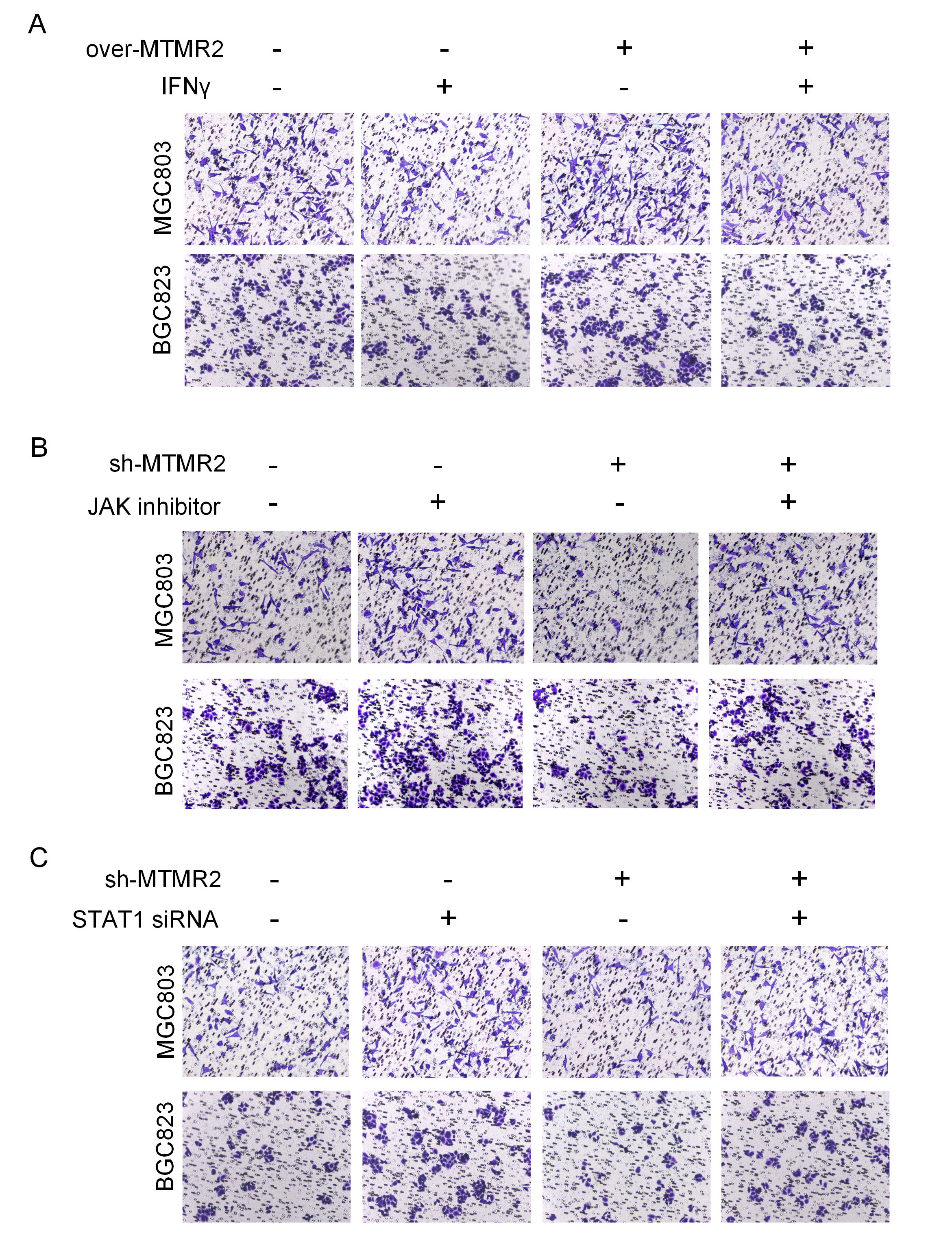


Figure S10. **Representative images of matrigel transwell invasion of GC cells.** (A) Representative images of matrigel transwell invasion assay for over-MTMR2 GC cells treated with or without IFNγ (50 ng/mL), a main inducer of STAT1 activation; **(B)** Representative images of matrigel transwell invasion assay for sh-MTMR2 GC cells treated with or without STAT1 siRNA (50 nmol/L), a specific inhibitor of STAT1 activation; **(C)** Representative images of matrigel transwell invasion assay for sh-MTMR2 GC cells treated with or without JAK inhibitor I (15 ng/mL), an inhibitor of JAK1/2.


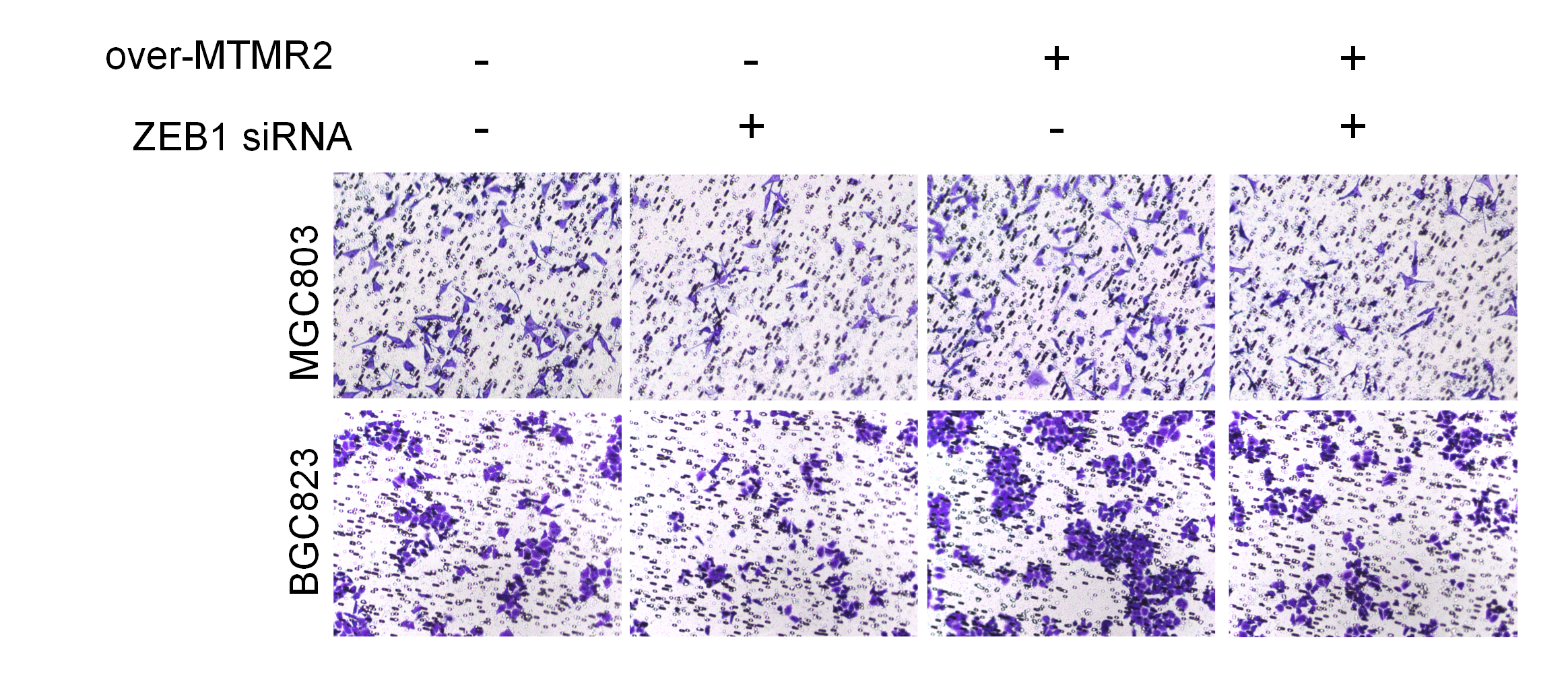


Figure S11. **Representative images of matrigel transwell invasion assay for sh-MTMR2 GC cells treated with or without ZEB1 siRNA (50 nmol/L).**


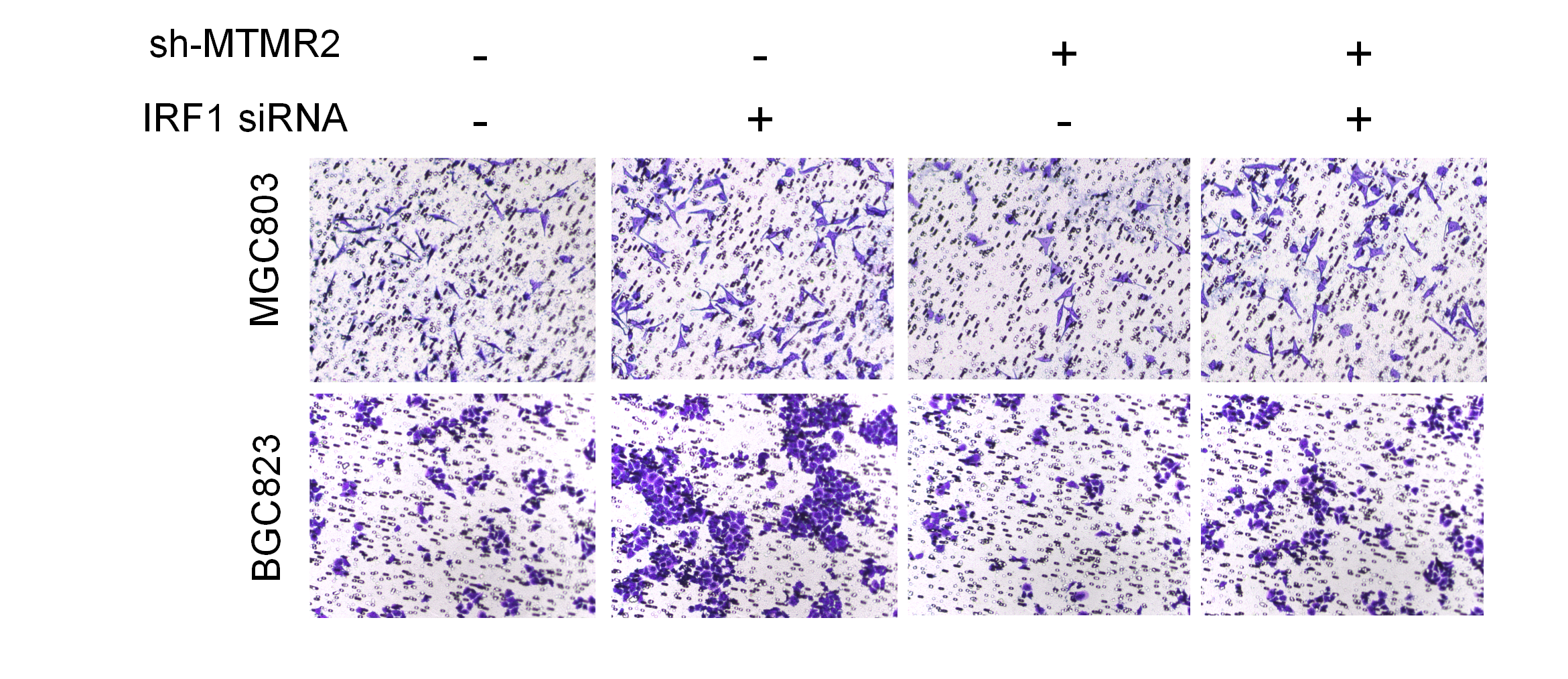


Figure S12. **Representative images of matrigel transwell invasion assay for sh-MTMR2 GC cells treated with or without** **IRF1 siRNA (50 nmol/L).**
